# Metabolomic Characterization of a cf. *Neolyngbya* Cyanobacterium from the South China Sea Reveals Wenchangamide A, a Lipopeptide with In Vitro Apoptotic Potential in Colon Cancer Cells

**DOI:** 10.3390/md19070397

**Published:** 2021-07-16

**Authors:** Lijian Ding, Rinat Bar-Shalom, Dikla Aharonovich, Naoaki Kurisawa, Gaurav Patial, Shuang Li, Shan He, Xiaojun Yan, Arihiro Iwasaki, Kiyotake Suenaga, Chengcong Zhu, Haixi Luo, Fuli Tian, Fuad Fares, C. Benjamin Naman, Tal Luzzatto-Knaan

**Affiliations:** 1 Li Dak Sum Yip Yio Chin Kenneth Li Marine Biopharmaceutical Research Center, Department of Marine Pharmacy, College of Food and Pharmaceutical Sciences, Ningbo University, Ningbo 315800, China; dinglijian@nbu.edu.cn (L.D.); gaurav.patial945@gmail.com (G.P.); lishuang9892@163.com (S.L.); heshan@nbu.edu.cn (S.H.); yanxiaojun@nbu.edu.cn (X.Y.); 2 Department of Marine Biology, Leon H. Charney School of Marine Sciences, University of Haifa, Haifa 31905, Israel; daharon1@univ.haifa.ac.il; 3 Department of Human Biology, Faculty of Life Sciences, University of Haifa, Haifa 31905, Israel; rbar-shal@univ.haifa.ac.il (R.B.-S.); ffares@univ.haifa.ac.il (F.F.); 4 Department of Chemistry, Keio University, 3-14-1, Hiyoshi, Kohoku-ku, Yokohama, Kanagawa 223-8522, Japan; b8213011@gmail.com (N.K.); a.iwasaki@chem.keio.ac.jp (A.I.); suenaga@chem.keio.ac.jp (K.S.); 5 Key Laboratory of Medicinal and Edible Plant Resources of Hainan Province, Hainan Vocational University of Science and Technology, Haikou 571126, China; zhu1447262684@163.com (C.Z.); hluo@hvust.edu.cn (H.L.); ftian@imu.edu.cn (F.T.)

**Keywords:** metabolomics, secondary metabolites, natural products, cyanobacteria, *Neolyngbya*, anticancer, drug discovery, South China Sea, wenchangamide

## Abstract

Metabolomics can be used to study complex mixtures of natural products, or secondary metabolites, for many different purposes. One productive application of metabolomics that has emerged in recent years is the guiding direction for isolating molecules with structural novelty through analysis of untargeted LC-MS/MS data. The metabolomics-driven investigation and bioassay-guided fractionation of a biomass assemblage from the South China Sea dominated by a marine filamentous cyanobacteria, cf. *Neolyngbya* sp., has led to the discovery of a natural product in this study, wenchangamide A (**1**). Wenchangamide A was found to concentration-dependently cause fast-onset apoptosis in HCT116 human colon cancer cells in vitro (24 h IC_50_ = 38 μM). Untargeted metabolomics, by way of MS/MS molecular networking, was used further to generate a structural proposal for a new natural product analogue of **1**, here coined wenchangamide B, which was present in the organic extract and bioactive sub-fractions of the biomass examined. The wenchangamides are of interest for anticancer drug discovery, and the characterization of these molecules will facilitate the future discovery of related natural products and development of synthetic analogues.

## 1. Introduction

Cyanobacteria have been shown to be prolific producers of structurally diverse natural products with a wide range of ecological and pharmacological activities [1,2,3]. Many discovered marine natural products have gone through clinical trials and even been accepted by regulatory agencies as drugs, and these include several antibody-drug conjugates that use a dolastatin/symplostatin marine cyanobacterium natural product derivative as an anti-cancer “warhead” [4]. Other cyanobacterial natural products have been advanced in anticancer drug discovery programs at the preclinical stage by means of total synthesis, medicinal chemistry analogue development, and pharmacological characterization of their mechanisms of action. Some notable lead molecules from cyanobacteria include the apratoxins, carmaphycins, coibamides, curacins, and largazoles, among others [1,2,3]. It is generally understood that the secondary metabolism of cyanobacteria, while energetically taxing, must serve some (often unknown) ecological function for the organisms. This has been demonstrated in a few specific cases, e.g., in the upregulation of microcystins production by some cyanobacteria in response to predation by grazers [5,6]. Filamentous cyanobacteria have also been reported to contain genetic information for biosynthesis of natural products comprising up to 20% of the genome, and even surpassing that in the example of some *Moorena* species, further supporting the importance to the organisms of this biosynthetic capacity on an evolutionary time scale [7]. However, it can be quite challenging to obtain or maintain filamentous cyanobacteria in axenic laboratory cultures, as well as perform molecular biology experiments with them [8]. A number of laboratory culture conditions is also understood to greatly impact not only the growth and survival of cyanobacteria, but also the associated natural product biosynthesis [9]. Accordingly, a majority of natural product chemicals reported historically from these organisms have come from larger environmental collections, or assemblages. A meta-analysis of all secondary metabolites reported from marine and microbial sources between 1941 and 2015 revealed that the chemistry of these samples is relatively source-specific, with the majority of cyanobacterial natural products being structurally dissimilar from those of all other producers examined [10].

The taxonomy of many documented filamentous cyanobacteria has come into question in the post-genomics era, and this is especially true for the *Lyngbya*-like and *Phormidium*-like morphotype [11,12,13]. For example, *Phormidium* is formally accepted as a part of the family *Oscillatoriaceae*, but it still appears in some literature reports and databases under *Phormidiaceae* (the *Phormidium*-like family) following previous taxonomic assignment and reclassification [14,15]. The genus *Phormidium* once comprised some 200 species; however, about 90% of these organisms have been redistributed into other genera, such as *Lyngbya*, and even different families in the order Oscillatoriales, including both *Oscillatoriaceae* and *Phormidiaceae*, after molecular characterization studies in recent years [16,17,18]. The members of genus *Lyngbya* have also been re-evaluated and revised several times [14]. After molecular characterization, several newly formed genera have emerged for organisms previously described as members of *Lyngbya*, notably including *Leptolyngbya*, *Moorena*, and *Okeania* [12,13,19,20]. More recently, the new genus *Neolyngbya* has also been created for several newly described *Lyngbya*-like organisms [21]. Despite having a reported biotechnological potential for drug discovery and development, only one new natural product has yet been reported from assemblages with *Neolyngbya*, namely the neurotoxic sesquiterpenoid eudesmacarbonate [22,23]. *Neolyngbya* organisms have not been previously reported in the South China Sea. Meanwhile, the South China Sea is home to a vastly understudied biodiversity of marine filamentous cyanobacteria [24,25]. This biodiversity resource has been largely under-examined, especially when compared to the vast chemical study of other types of microorganisms in China (actinomycetes, fungi, etc.) [26].

Metabolomics is useful for the large-scale analysis of molecules within a biological sample [27]. In recent years, this field has taken a central role in many natural product research programs, especially for studying the chemical space and diversity using both untargeted and targeted metabolomics [28]. Untargeted metabolomics allows for the generation of a broad overview of the chemical diversity in even a complex extract. This can also be used for the comparative analysis of multiple samples, or various treatment conditions, to identify potential characteristic and chemical markers. In contrast, targeted metabolomics is useful when the focus can be specified to a single compound of interest or a set of pre-determined molecules for further qualitative and quantitative analysis. Mass spectrometry-based metabolomics in the past decade has shown immense utility in the field of natural product discovery, and has yielded major impacts, mainly because of the accuracy, sensitivity, speed, and robustness of these methods, along with newly developed cutting-edge downstream platforms for data analysis [29,30,31,32,33,34,35,36]. These platforms have been made to provide structural information based on the fragmentation patterns of each molecule, allowing for the comparison of each with other known and unknown compounds in spectrometric libraries, natural product databases, and public or private collections of raw data. Altogether, this has facilitated the characterization of putative structures based on a similarity between the fragmentation of different compounds, minimized the rediscovery of known structures by virtual dereplication, and allowed for a more efficient discovery of new natural products and new chemical scaffolds prior to the isolation and characterization effort [29,30,31,32,33,34,35,36].

**Figure 1:**
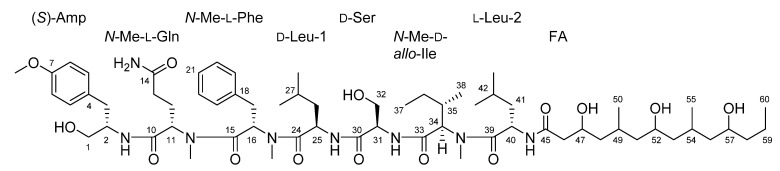
Structure and numbering scheme of wenchangamide A (**1**).

In this study, a metabolomics-based approach was used to explore the chemistry of a cf. *Neolyngbya* sp. environmental collection and characterize novel natural product chemistry. Moreover, the concurrent bioactivity-guided fractionation of this extract was expected to yield pure compounds produced with potential anti-cancer effects, as evaluated in vitro using an immortalized colorectal cancer cell line. Reported herein is the chemical and biological exploration of an environmental collection from the South China Sea that is dominated by a marine filamentous cyanobacteria, cf. *Neolyngbya* sp. This report details the characterization of the microbiome, metabolome, and associated pharmacology that allowed for the directed isolation of a new bioactive natural product, wenchangamide A (**1**; Figure 1). The structure elucidation and investigation of this molecule as a potential anticancer drug lead is also described, along with the expansion of this class of compound to include a new proposed bioactive analogue based on available metabolomics and bioassay testing data.

## 2. Results and Discussion

### 2.1. Sample Evaluation

**Figure 2:**
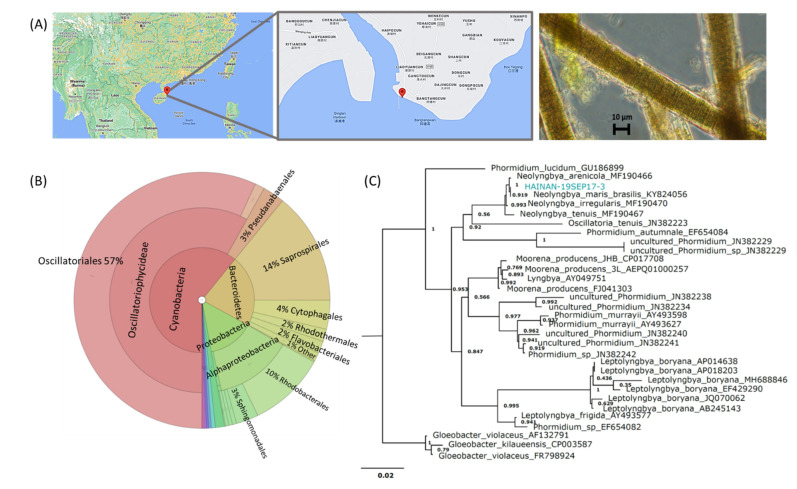
Sample information. (**A**) Collection site and morphology of HAINAN-19SEP17-3. (**B**) Microbiome analysis; the order Oscillatoriales contains the families *Oscillatoriaceae* and *Phormidiaceae*, and this higher taxonomy is presented here to avoid confounding basionyms within these two. (**C**) Phylogeny of the environmental assemblage dominated by cf. *Neolyngbya* sp. from the South China Sea that was evaluated in this study. Map generated with Google Earth. Taxonomy and phylogeny were evaluated using Silva and EMBL-EBI databases. *Gleobacter* was used as the outgroup.

An environmental sample of marine filamentous cyanobacteria, HAINAN-19SEP17-3, was collected near Wenchang, Hainan, China. Based on colonial morphology and light microscopy, the sample was initially classified as cf. *Neolyngbya* sp. (Figure 2). To validate this and determine the microbiome composition, a portion of the sample was analyzed by 16S rRNA gene sequencing using universal PCR primers, and this further supported the genetic identity of the predominant biomass as cyanobacteria categorized under *Phormidiaceae* (57%; certainly includes basionyms in *Oscillatoriaceae*) along with additional associated microbes from *Bacteroidetes* (22%), *Proteobacteria* (14%), and others at a lower abundance (Figure 2). The higher taxonomic order Oscillatoriales is presented for the majority of the cyanobacterial 16S gene sequence data in Figure 2 to avoid confounding basionyms that occur within its members, i.e., parts of the families *Oscillatoriaceae* and *Phormidiaceae*. The 16S gene sequence V3-V4 amplicon of the organism that dominates this consortium was found to clade with *Neolyngbya*. *Neolyngbya* is a recently described genus of the family *Oscillatoriaceae*, and was established from the *Lyngbya*-like morphotype that has historically also been a misclassification for some *Phormidium* organisms [16,17,21]. There is great difficulty in growing axenic cultures of cyanobacteria; therefore, it is important to refer to the collected consortia as a whole. While several studies demonstrated that the microbiome of cyanobacteria is relatively stable between environmental samples and non-axenic cultures (mainly *Proteobacteria* and *Bacteroidetes*) [37], little is known about the microbiome associated with *Lyngbya*-like and *Phormidium*-like organisms [38].

**Figure 3:**
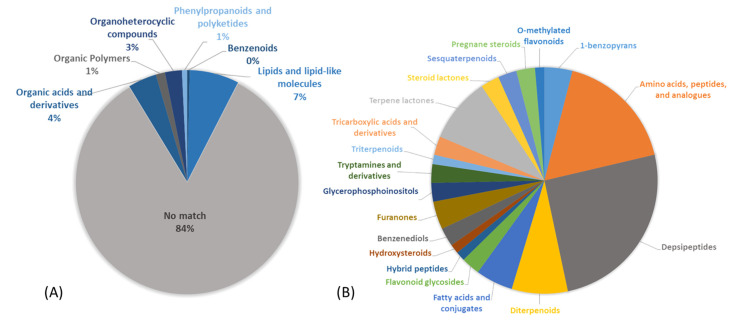
Chemical space of the organic extract of cf. *Neolyngbya* sp. HAINAN-19SEP17-3 as evaluated by data-dependent LC-MS/MS. Samples were analyzed via the GNPS platform using NAP, Dereplicator and MolNetEnhancer workflows to yield putative annotations of (**A**) SuperClasses and (**B**) SubClasses of annotated molecular features based on observed fragmentation patterns.

An LC-MS/MS untargeted metabolomic approach [28] was utilized to overview the chemical potential of the prioritized South China Sea cf. *Neolyngbya* sp. sample. Feature detection and annotation analyses were done using the Global Natural Products Social (GNPS) Molecular Networking platform. This method aligns the fragmentation patterns obtained by MS/MS against various spectrometric databases and allows for the putative annotation of structural characteristics and chemical classifications [33,36]. Nearly 750 molecular features were present in the initial evaluation of this sample; however, reported cyanobacterial specialized metabolites were not able to be detected. Some common pigments (mainly chlorophylls and breakdown products thereof) were annotated in the dataset. Together, these data highlighted the potential for discovery of novel compounds and, at the same time, allowed ubiquitous pigment molecules to be avoided in the isolation procedure. Furthermore, most of the chemistry had no match to any known structure in the spectrometric libraries (84%), yet some had putative annotations to general chemical classes (5 super-classes; Figure 3A), based on the associated fragmentation patterns. The subset of classified molecules were further delineated into 19 putative chemical subclasses (Figure 3B) that highlight the chemical diversity and discovery potential of this complex extract. The main prevalent classes that were detected and annotated include peptides (42%) and terpenoids (17%). Though databases on such molecules are largely incomplete, or hard to access, these molecular families are known to contain many types of bioactive natural products [39,40,41]. Nonribosomal peptides are a diverse group of natural products that have complex chemical structures and a vast array of bioactivity potentials as anticancer, anti-parasitic, anti-fungal, and cytotoxic agents, protease inhibitors and more [39]. The structures of natural products resulting from non-ribosomal peptide synthetase (NRPS) biosynthesis can be linear or cyclic, possess typical and/or unusual amino acids, and may even be hybridized with modules from polyketide synthase (PKS) genes. NRPS and PKS biosynthetic gene clusters are mostly common in bacteria, and many such hybridized biosynthetic mechanisms have been uniquely found in cyanobacteria or are rarely described from other organisms [39,42]. The metabolomic annotation of unknown peptides, depsipeptides and derivatives from the cyanobacteria sample here studied was accordingly encouraging for the potential to discover new bioactive molecules.

### 2.2. Inhibition Activity on Human Colon Cancer Cells In Vitro

The inhibitory effect of the organic extract and fractions of cf. *Neolyngbya* sp. HAINAN-19SEP17-3 were evaluated using HCT116 human colorectal cancer cells (Figure S23A). This allowed for the targeted discovery of new bioactive natural products akin to a published method [43]. Cells were treated for 24 h and analyzed using an XTT cell viability assay to detect fast-acting fractions and compound constituents [44]. It is understood that extended duration exposure (e.g., to 48 or 72 h) will typically increase the observed efficacy or potency of cytotoxicity due to the relatively prolonged accumulation of dead cells. While the crude extract was not cytotoxic at the concentrations tested (200 and 400 μg/mL) in this 24 h experiment, fraction C demonstrated high potency (94–97% mortality) in treated cells versus untreated at both concentrations tested (Figure S23A). After further separation into 6 sub-fractions, a more marked concentration-dependent activity was observed for C3 (Figure S23B). Additional chromatography yielded sub-fractions that were also shown to act concentration-dependently, i.e., C3–5 and C3–7 (Figure S24). While the active fraction C3–7 was observed to be an impure mixture of compounds, fraction C3–5 was found to be a pure molecule (**1**) that was active in this in vitro test model (24 h IC_50_ = 38 μM), and noticeably active even after only 8 h of treatment (Figure S25). This sample was thus evaluated further.

**Figure 4:**
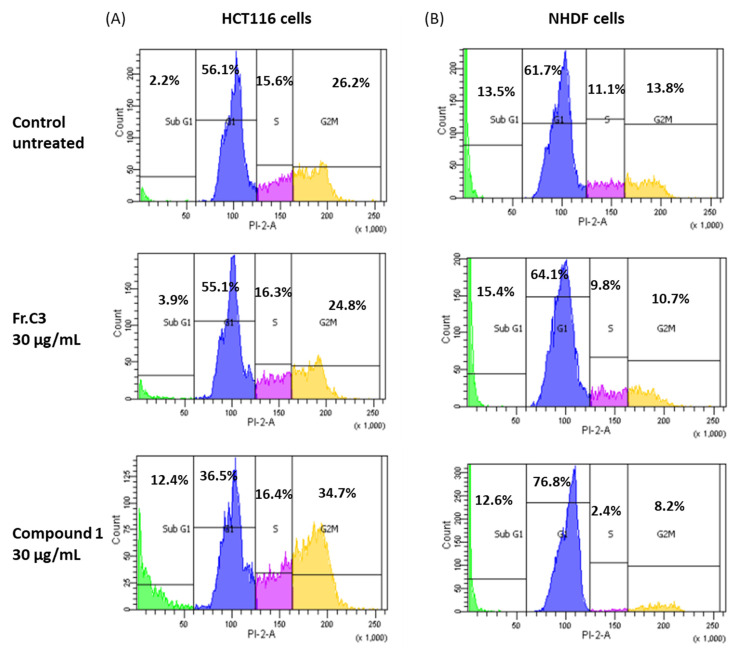
In vitro effects of fraction C3 and compound **1** on cell cycle progression after 24 h treatment. Distribution of (**A**) HCT116 human colon cancer cells and (**B**) NHDF normal human dermal fibroblasts at the different cell cycle phases as determined by FACS.

To clarify the cell viability decrease following 24 h treatment with C3 and **1** (30 µg/mL), cell cycle distribution analysis was examined. A FACS analysis demonstrated that treatment with C3 and **1** resulted in the accumulation of cells in the sub-G1 phase of the cell cycle at 3.9% and 12.4%, respectively, compared to 2.2% in the untreated (control) cells (Figure 4A). Furthermore, the cells were observed to be accumulating at the G2/M phase following treatment with **1** (34.7% vs. 26.2% in the control), indicating suppression of cell proliferation. Normal, non-cancerous colon cell lines are unavailable. However, the same pattern of cell cycle arrest was not observed when the samples were tested in normal human dermal fibroblasts (NHDF; Figure 4B). 

**Figure 5:**
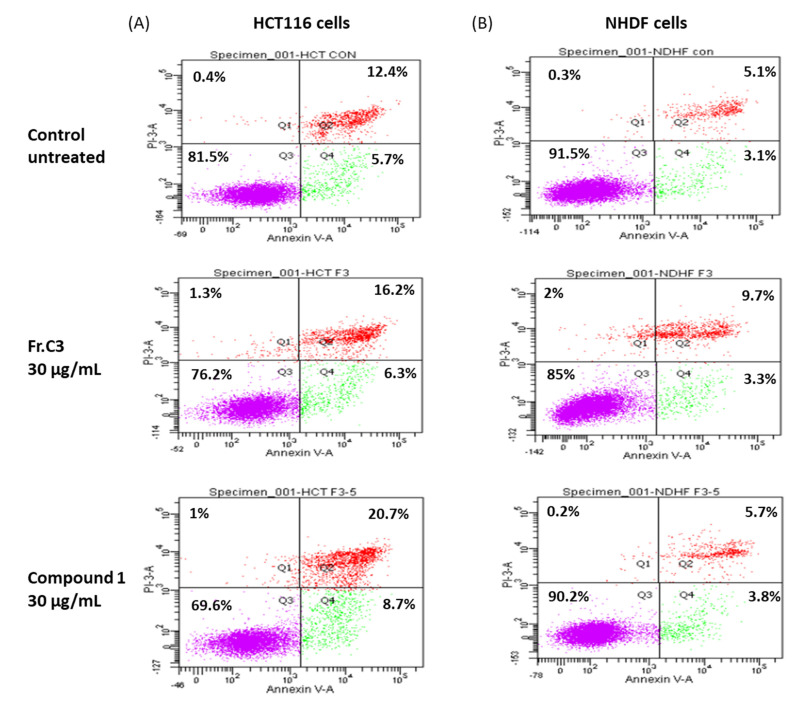
Annexin-V/PI double staining and flow cytometry evaluation of mechanistic in vitro cytotoxicity of fraction C3 and compound **1** after 24 h treatment of (**A**) HCT116 human colon cancer cells and (**B**) NHDF normal human dermal fibroblasts. For each plot, the lower left quadrant (Q3) represents viable cells, the upper left quadrant (Q1) indicates necrotic cells, the lower right quadrant (Q4) denotes early apoptotic cells, and the upper right quadrant (Q2) represents necrotic or late apoptotic cells.

The cell cycle arrest at the G2/M phase accompanied by an accumulation in the sub-G1 phase observed due to treatment with C3 or **1** is suggestive of apoptotic cell death, since this has been reported previously for human colon cancer cells [45]. In order to confirm this hypothesis, HCT116 cells were treated with 30 µg/mL C3 or **1** for 24 h, stained with FITC labeled Annexin-V and PI, and analyzed by flow cytometry (Figure 5A). The results indicated an increase of approximately 4.4% in apoptotic cells (Q2 + Q4) following treatment with C3, and about 11.3% after exposure to **1**. Annexin/PI double staining analysis of NHDF cells in vitro showed a similar increase in accumulation of apoptotic cells after treatment with fraction C3, of about 4.8%, but a much smaller increase following treatment with compound **1**, of about 1.3%, in comparison to untreated cells (Figure 5B).

### 2.3. Natural Product Structure Elucidation

**Figure 6:**
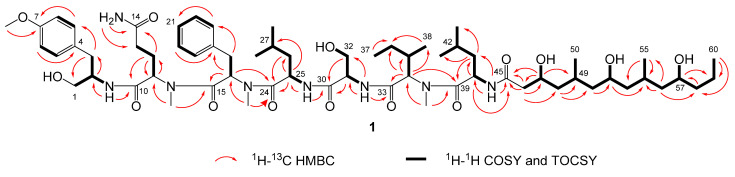
Selected correlations used to determine the planar structure of wenchangamide A (**1**). Red single-sided arrows represent cross peaks from the ^1^H-^13^C HMBC spectrum. Black bolded bonds show protons correlated in the ^1^H-^1^H COSY and TOCSY spectra.

Compound **1** was obtained as a white powder and assigned the molecular formula C_64_H_106_N_8_O_14_ based on a sodium adduct peak in the HRESIMS spectrum at *m*/*z* 1233.7748 [M + Na]^+^ (calcd. for C_64_H_106_N_8_O_14_Na^+^, 1233.7721). This formula indicated that **1** possessed 16 degrees of unsaturation. The ^1^H and ^13^C NMR data of **1** (Table 1) were suggestive of a lipopeptide scaffold with seven sets of signals characteristic of amino acid α protons, as well as two aromatic rings, two oxygenated methylenes, three oxygenated methines, one methoxy and three *N*-methyl groups, along with many alkyl moieties and eight amide carbonyls. The region measured from *δ*_H_ 3.8 to 4.9 ppm had sufficient peak resolution to nucleate seven amino acid and derivative substructures that were able to be constructed using 1D and 2D NMR data. For example, an “α proton” signal at *δ*_H_ 3.91 (H-2) was connected to a carbon at *δ*_C_ 52.4 (C-2) with the evidence of a peak in the ^1^H-^13^C HSQC spectrum. After examination of the ^1^H-^1^H COSY spectrum and HSQC data, this methine was determined to be adjacent to an oxygenated methylene group, CH_2_-1 (*δ*_C_ 62.7, *δ*_H_ 3.35), and a benzylic methylene group, CH_2_-3 (*δ*_C_ 35.6, *δ*_H_ 2.77 and 2.53). The assignment of the aromatic ring connected to C-3 was completed by further inclusion of long-range coupling data obtained from the ^1^H-^13^C HMBC spectrum. As shown in Figure 6, this *para*-methoxy-substituted phenyl group was characterized by correlations observed between H_2_-3 and C-5/9, H-5/9 and C-7, H_3_-7-*O*-Me and C-7, as well as H-6/8 and C-4. The planar structure of this subunit was thus established as 2-amino-3-(4-methoxyphenyl)propan-1-ol; “Amp”. 

**Figure 7:**
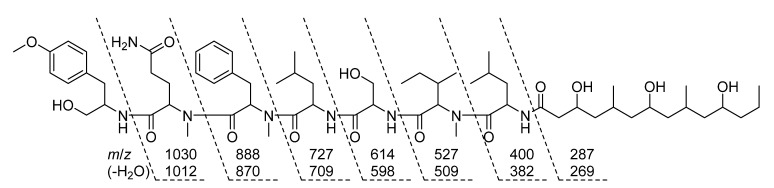
Selected MS/MS fragmentation ions observed that supported the amino acid and derivative residue sequence in the planar structure of wenchangamide A (**1**).

Much of the remaining NMR data for **1** could be further assigned to a series of standard or *N*-methyl amino acid residues that were determined by similar methods as for the Amp group, including two Ile residues, an *N*-Me-Gln, *N*-Me-Phe, *N*-Me-Ile, and Ser. Several of the aliphatic groups had partially overlapping signals in the ^1^H NMR spectrum, e.g., H_2_-12 (*δ*_H_ 1.97 and 1.61) and H-42 (*δ*_H_ 1.61), as well as H_2_-13 (*δ*_H_ 1.90 and 1.83) and H-35 (*δ*_H_ 1.9), which complicated their assignment using NMR data from the COSY or even ^1^H-^1^H TOCSY spectra. However, these groups were differentiated and assigned conclusively by the resolution of their corresponding signals in the HSQC and HSQC-TOCSY spectra, e.g., C-12 (*δ*_C_ 23.8) and C-42 (*δ*_C_ 24.3), as well as C-13 (*δ*_C_ 31.3) and C-35 (*δ*_C_ 32.6). Since the signals from TOCSY and HSQC-TOCSY result from extended or even complete ^1^H-^1^H spin system couplings, the signals observed from the well-resolved region (*δ*_H_ 3.8 to 4.9 ppm) in the f2 dimension were sufficient to support the assignment of the structural subunits described above. Each of the three *N*-methyl groups was able to be assigned to a defined amino acid residue based on correlations observed in the HMBC spectrum, i.e., from H_3_-11-*N*-Me (*δ*_H_ 2.42) to C-11 (*δ*_C_ 56.0), H_3_-16-*N*-Me (*δ*_H_ 2.89) to C-16 (*δ*_C_ 54.0), and H_3_-34 *N*-Me (*δ*_H_ 2.94) to C-34 (*δ*_C_ 59.9). Amide NH protons were similarly able to be assigned by correlations observed in the COSY and TOCSY spectra, i.e., from 2-NH (*δ*_H_ 7.27) to H-2, 25-NH (*δ*_H_ 8.04) to H-25 (*δ*_H_ 4.49), 31-NH (*δ*_H_ 7.53) to H-31 (*δ*_H_ 4.23), and 40 NH (*δ*_H_ 8.15) to H-40 (*δ*_H_ 4.72). As further shown in Figure 6, the sequence of amide or “peptide” bonds was able to be deduced from the HMBC correlations observed between *N*-Me, NH, and “α proton” signals to the carbonyl of the adjacent residue. The sequence order of these structural subunits was further supported by characteristic amide bond “y” fragmentation masses that were detected in the MS/MS spectrum of **1** (Figure 7).

All of the NMR data that remained unassigned was proposed to result from a poly-hydroxylated fatty acid moiety (FA), since this corresponded to three oxygenated methines, six downfield methylenes, and three alkyl methyl groups and one carbonyl. Due to diagnostic HMBC correlations from both H-40 and 40-NH to the remaining unassigned carbonyl (*δ*_C_ 171.0; C-45), the attachment point for this structural subunit was able to be assigned to the nitrogen of the Leu-2 residue. Further HMBC correlations to C-45 were observed from a deshielded methylene (*δ*_H_ 2.22 and 2.13, *δ*_C_ 43.8; CH_2_-46) and a more deshielded, oxygenated, methine (*δ*_H_ 3.87, *δ*_C_ 65.7; CH-47). This allowed for the generation of a growing alkyl carbon chain that was able to be extended by COSY correlations, i.e., from CH-47 to CH_2_-48 (*δ*_H_ 1.23 and 0.92) and then CH-49 (*δ*_H_ 1.76), as well as signals in the HMBC spectrum, including from H_2_-46 to C-48 (*δ*_C_ 45.2) and H-47 to C-49 (*δ*_C_ 25.6). CH-49 was connected to and had a COSY correlation with a methyl group (*δ*_H_ 0.82, *δ*_C_ 20.3; CH_3_-50). This PKS-like subunit, –CH_2_–(CH–OH)–CH_2_–(CH–CH_3_)–, was found to repeat two more times in the linear alkyl chain of the FA moiety, and terminated the molecule with an alkyl methyl group (*δ*_H_ 0.85, *δ*_C_ 14.2; CH_3_-60) adjacent to a penultimate methylene unit (*δ*_H_ 1.35 and 1.26, *δ*_C_ 18.6; CH_2_-59). In sum, this yielded the planar structure of **1** as shown in Figure 6. Compound **1** is a new natural product, here assigned the trivial name wenchangamide A due to the location of the geographical collection site that yielded this discovery.

**Figure 8:**
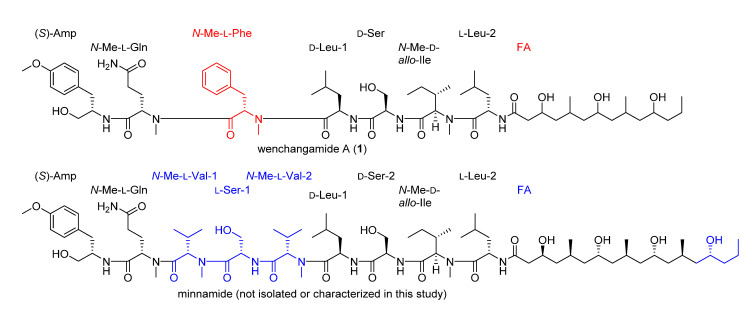
Structural comparison of wenchangamide A (**1**) and minnamide. Shared structural motifs are drawn in black. Differences in **1** are highlighted in red. Differences in minnamide are highlighted in blue. Configurations from the shared part of the FA residue in **1** are hypothesized to match those of minnamide.

The structure of **1** has many features that resemble minnamide A, a cyanobacterial natural product recently reported from a sample of *Okeania hirsuta* that was collected in Minna island, Okinawa Prefecture, Japan [46]. However, noteworthy differences in the structures (Figure 8) include a different length polypeptide core scaffold, where minnamide A has an *N*-Me-Val–Ser–*N*-Me-Val moiety instead of the *N*-Me-Phe group present in **1**, as well as a longer fatty acid tail that contains an additional PKS-like repeating unit described above (repeats 3x in **1** and 4x in minnamide A). Accordingly, the molecular weight of minnamide A is 238 Da higher than that of **1**, and these molecules have significantly different MS/MS spectra due to the multiple structural differences. However, the hydrolysis of an aliquot of **1**, and subsequent analysis by chiral HPLC along with standard compounds, supported the assignment of the same configuration for all shared amino acid residues and derivatives from minnamide A, specifically (*S*)-Amp, *N*-Me-l-Gln, d-Leu-1, d-Ser, *N*-Me-d-*allo*-Ile, and l-Leu-2. Comparison of the NMR data obtained for **1** in pyridine-*d*5 (see Supplementary Table S1) with published values for minnamide further supported these assignments [46]. The *N*-Me-Phe residue (present in **1** and absent in minnamide A) was determined to be that of the l form by the same protocol. It is hypothesized that the configuration of the repeating PKS-like subunits of the fatty acid chain in **1** match with those reported for minnamide A; however, this has not been established empirically in the present study. In total, this information was used to assign the absolute configuration of the peptide core scaffold of **1** as presented in Figure 1 and Figure 8.

### 2.4. Additional Structure Hypothesis Generation

**Figure 9:**
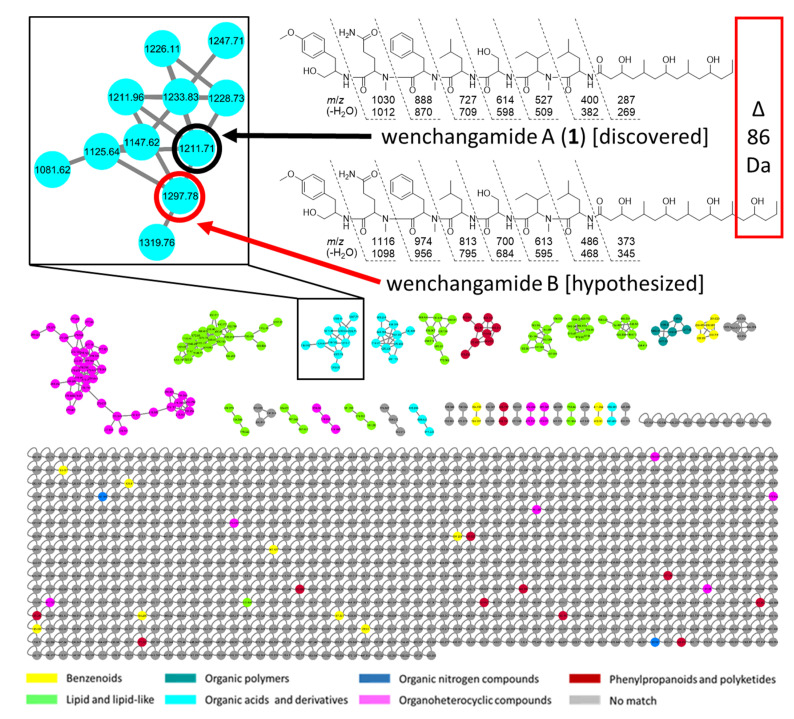
GNPS-based LC-MS/MS molecular network analysis of fraction C3 and active sub-fractions. Enlarged network highlights the structural similarity based on MS/MS fragmentation patterns of the discovered molecule **1** (wenchangamide A; node circled in black) and the proposed analogue (wenchangamide B; circled in red).

The GNPS-produced LC-MS/MS molecular network highlights molecules with a potential structural similarity as “molecular families”, based on fragmentation patterns. The cluster that contained wenchangamide A (**1**) suggested the presence of further analogue molecules in the extract. One of these compounds presented an *m*/*z* value of 1297, which is 86 Da higher than that of **1** in the same experiment. Upon closer examination of the MS/MS spectra and fragmentation ions related to the “y” ions produced from amide bond backbone cleavages, it was determined that the entire difference of 86 Da between the *m*/*z* 1297 molecule and **1** was located on the FA residue. Since this same mass difference corresponds to one additional repeating unit of a PKS-like moiety present 3x in **1,** akin to the 4x in minnamide A, the corresponding “hybrid” molecule, wenchangamide B, is here hypothesized (Figure 9). While the structure of this molecule contains the same FA moiety from minnamide A, it has the same polypeptide core as **1**, and is accordingly assigned the trivial name, wenchangamide B. Although fragmentation data from mass spectrometry cannot distinguish between configurational isomers of peptides, and even some constitutional isomers (e.g., Ile, Leu, and *N*-Me-Val), the structure of wenchangamide B is proposed as drawn, based on biosynthetic logic. This molecule was not able to be isolated in pure form in sufficient quantity for empirical structural characterization in this study, and is suggested for targeted isolation, structure elucidation, and more accurate pharmacological characterization in future research. This strategy of structure proposal based on MS/MS fragmentation analysis and molecular networking for compounds beyond isolation in the initial study, later followed by targeted isolation or synthesis for confirmation and structure-activity relationship (SAR) study, has been recently exemplified by Gerwick, Luesch, and coworkers in the expansion of the cyanobacterial natural product family of doscadenamides [47,48,49]. 

The reported activities of minnamide and **1** have been demonstrated in discrete cell lines, with different conditions, and using alternative temporal end points for measurement. In terms of activity, minnamide A was reported to be a potent inhibitor of HeLa cells that led to necrosis in a 72 h incubation assay (IC_50_ 0.17 μM); it was also suggested to act via the generation of lipid ROS facilitated by specific metal ions including copper and manganese [46]. Wenchangamide B remains of particular interest because the parent subfraction (C3-7) was here found to inhibit HCT116 cells in vitro (Figure S24). Future research on new natural products and synthetic analogues may contribute relatable SAR data for the growing class of cytotoxic minnamide and wenchangamide lipopeptides.

## 3. Materials and Methods

### 3.1. General Experimental Procedures

Analytical separations were performed on a Waters ACQUITY UPLC instrument employing a UPLC Kinetex C18 column (1.7 μm, 2.1 × 50 mm, Phenomenex) and an HPLC Kinetex C18 column (5 μm, 5 × 250 mm, Phenomenex), respectively, combined with a Waters 2998 photodiode array detector (PDA) (Waters, Milford, MA, USA). Medium pressure liquid chromatography (MPLC) was carried out on a Biotage-Isolera One system (SE-751 03 Uppsala, Sweden) equipped with a YMC-Pack ODS-A column (500 mm × 50 mm, 50 μm, YMC, Tokyo, Japan). All LC/MS data were obtained on a Phenomenex Kinetex C18 analytical column (1.7 μm, 2.1 × 50 mm, Phenomenex) using an Agilent HPLC equipped with a Bruker Maxis impact QTOF system mass spectrometer. Chromatographic analyses for configuration analysis were performed using an HPLC system consisting of a pump (model PU-2080, JASCO, Easton, MD, USA) and a UV detector (model UV-2075, JASCO). The NMR data were recorded using standard pulse programs on a Bruker AVANCE NEO 600 spectrometer equipped with a 5 mm inverse detection triple resonance (H-P/C/N-D) QCI 600S3 cryoprobe, capable of applying z-gradients. The chemical shifts were calibrated relative to the residual solvent peak in DMSO-*d*_6_ (*δ*_H_ 2.50 and *δ*_C_ 39.52). High-resolution electrospray ionization mass spectra (HRESIMS) were measured on an Agilent (Santa Clara, CA, USA) 6545 Q-TOF instrument. Optical rotations were measured with a JASCO P-2000 automatic polarimeter.

### 3.2. Cyanobacterial Collection and Taxonomy

The biomass of the environmental sample of marine filamentous cyanobacteria used in this research was collected by hand by several of the co-authors on 17 September 2019 from the intertidal zone (0–2 m deep water) near Bangtang Bay, Wenchang District, Hainan Province, China (N 19°31′43.9′′, E 110°51′02.7′′). A voucher specimen for this organism was encoded as HAINAN-19SEP17-3 and deposited in the repository of the Department of Marine Pharmacy, Ningbo University (available from C.B.N., Ningbo, China). A small sample of this material was preserved in RNAlater solution for molecular analysis, and the rest was directly frozen at −18 °C for transportation to the lab and storage in the same condition until the time of chemical extraction. The majority of the biomass present was tentatively identified as a marine filamentous cyanobacterium belonging to the *Lyngbya*-like and *Phormidium*-like morphotype based on its macroscopic colonial appearance and morphological features observed under light microscopy (Figure 2). The taxonomy of this organism was further refined to a cf. *Neolyngbya* sp. by independent 16S rRNA gene sequencing at Beijing Genomics Institute, using universal bacteria PCR primers for the 16S-V3-V4 region and Operational Taxonomic Unit mapping using USEARCH. Data were visualized using the Krona Tools web browser [50]. Phylogenetic tree by neighbor joining was generated via SILVA [51].

### 3.3. Extraction and Isolation

The freeze-dried and powdered biomass (dry weight 600 g) of the above environmental collection was exhaustively extracted in 2:1 CH_2_Cl_2_/MeOH (8×). The extracts were dried under vacuum and then rinsed with H_2_O (3×) to remove residual sea salt, affording 3.1 g of an organic crude extract. This material was subjected to vacuum liquid chromatography (VLC) separation over normal-phase silica gel column chromatography (200–300 mesh) using a stepwise gradient (10% EtOAc/hexanes, 20% EtOAc/hexanes, 40% EtOAc/hexanes, 60% EtOAc/hexanes, 80% EtOAc/hexanes, 100% EtOAc, 10% MeOH/EtOAc, and 20% MeOH/EtOAc). The column was eluted to provide 8 fractions: A (200.9 mg), B (300.5 mg), C (316.4 mg), D (250.6 mg), E (150.9 mg), F (707.5 mg), G (506.7 mg), and H (259.6 mg). Fraction C was further separated by RP-18 MPLC with a 120 g Biotage SNAP Cartridge, KP-C18-HS, and a gradient solvent system (60% to 100% MeOH/H_2_O in 60 min) to generate subfractions C1–C6 with yields of 40 mg, 40 mg, 60 mg, 30 mg, 20 mg, and 70 mg, respectively. Subfraction C3 was additionally fractionated using a Waters ACQUITY UPLC equipment instrument equipped with a PDA detector on a reversed-phase Phenomenex Kinetex C18 column (5 μm, 5 × 250 mm, Phenomenex) using MeCN/H_2_O as a mobile phase, at a flow rate of 1 mL/min. The gradient program was 50–100% MeCN/H_2_O in 20 min with a linear gradient elution. The eluent was delivered to an automatic fraction collector for timed sampling every 0.75 min from 6.2 min to 15.2 min, and all the fractions were dried in glass tubes and weighed. In total, 12 fractions, Fr.C3–1−C3–12 were obtained from the subfraction C3 with the yields 1 mg, 6 mg, 1 mg, 2 mg, 3 mg, 2 mg, 3 mg, 1 mg, 1 mg, 1 mg, 2 mg, and 2 mg, respectively. Fraction C3–5, corresponding to a 3 mg sample collected around a UV 210 nm peak at *t*_R_ = 9.6, was found to contain the pure compound **1** after it was analyzed by LC-MS and 1D NMR.

### 3.4. Isolated Materials (New Natural Products)

*Wenchangamide A (**1**)*: white powder; [α${]}_{D}^{17}$ −315 (*c* 0.1, MeOH); UV (MeOH) *λ*_max_ (log *ε*) = 218 (3.92), 275 (3.04) nm; IR (KBr) ν_max_ 3350 (br), 2921, 2815, 2742, 1612, 1515, 1505, 1487, 1416, 1235 cm^−1^;for ^1^H NMR and ^13^C NMR data see Table 1; HR-ESI-MS [M + Na]^+^ *m/z* 1233.7748 (calcd. for C_64_H_106_N_8_O_14_Na^+^, 1233.7721).

### 3.5. Determination of the Absolute Configuration of Wenchangamide A (1)

Wenchangamide A (**1**, 1.0 mg) was treated with 6 M HCl (100 μL) for 24 h at 110 °C. The hydrolyzed product was evaporated to dryness for purification of the individual structural components. Using HPLC separation and a Cosmosil 5C_18_-PAQ column [(ϕ4.6 × 250 mm); flow rate, 1.0 mL/min; UV detection at 215 nm; solvent H_2_O], the components; Ser (*t*_R_ = 2.6), Leu and *N*-Me-Ile (*t*_R_ = 5.2), and *N*-Me-Phe (*t*_R_ = 12.3) were collected. In another HPLC separation using the Cosmosil 5C_18_-PAQ column [(ϕ4.6 × 250 mm); flow rate, 1.0 mL/min; UV detection at 215 nm; solvent 0.1% aqueous TFA], *N*-Me-Glu (*t*_R_ = 3.4) was obtained. In a final HPLC separation using the Cosmosil 5C_18_-PAQ column [(ϕ4.6 × 250 mm); flow rate, 1.0 mL/min; UV detection at 215 nm; solvent 40% aqueous MeOH], Amp (*t*_R_ = 3.7) was collected. 

Each hydrolyzed fraction, except for Amp, was dissolved in H_2_O and analyzed by chiral HPLC, and the retention times were compared to those of authentic standards. For this analysis, a DAICEL CHIRALPAK MA(+) column [(ϕ4.6 × 50 mm); flow rate 1 mL/min; detection, UV 254 nm; solvent 2.0 mM CuSO_4_, 2.0 mM CuSO_4_/MeCN = 95/5] was used. With 2.0 mM CuSO_4_ as a solvent, the retention times of *N*-Me-Glu and Leu hydrolyzed from **1** matched those of the authentic standards of *N*-Me-l-Glu (20.2 min; *N*-Me-d-Glu, 18.6 min), d-Leu (9.6 min) and l-Leu (18.7 min). With 2.0 mM CuSO_4_/MeCN = 95/5 as a solvent, the retention time of *N*-Me-Phe hydrolyzed from **1** matched that of the authentic standard of *N*-Me-l-Phe (19.8 min; *N*-Me-d-Phe, 16.5 min). Increased resolution was required for the Ser residue, and a series of two DAICEL CHIRALPAK MA(+) columns [(ϕ4.6 × 50 mm); flow rate 1 mL/min; detection, UV 254 nm; solvent 2.0 mM CuSO_4_] was used. The retention time of Ser hydrolyzed from **1** matched those of the authentic standards of d-Ser (2.5 min; l-Ser, 3.2 min).

For analysis of the Amp and *N*-Me-Ile residues, Marfey’s method was used to clarify the absolute configurations. To each isolated residue was added 1.0% l-FDLA acetone sol. (100 µL) and 1 M NaHCO_3_ (25 µL). The mixtures were heated at 80 °C for 3 min, cooled to room temperature, and neutralized with 1 M HCl. The products were analyzed by HPLC and the retention time was compared with those of authentic standards. A Cosmosil Cholester column [(ϕ4.6 × 250 mm); flow rate 1 mL/min; detection, UV 340 nm; solvent MeCN/H_2_O/TFA = 70/30/0.1] was used to evaluate the Amp derivatives. The retention time of Amp-l-FDLA from hydrolysate of **1** matched that of the authentic standard (*S*)-Amp-l-FDLA (t*_R_* = 4.9 min; (*S*)-Amp-d-FDLA, 5.6 min). A Cosmosil PBr column [(4.6 × 250 mm); flow rate 1 mL/min; detection, UV 340 nm; solvent MeCN/H_2_O/TFA = 55/45/0.1] was used to evaluate the *N*-Me-Ile derivatives. The retention time of *N*-Me-Ile-l-FDLA from hydrolysate of **1** matched that of the authentic standard *N*-Me-d-*allo*-Ile-l-FDLA (t*_R_* = 17.8 min; *N*-Me-l-Ile-l-FDLA 14.2 min; *N*-Me-d-Ile-l-FDLA 17.3min; *N*-Me-d-*allo*-Ile-d-FDLA 14.7 min).

### 3.6. LC−MS Analysis and Molecular Networking Generation

The crude extract and fractions A−H were dissolved in MeOH at 0.5 mg/mL. A 50 μL aliquot of each sample was injected via LC−MS/MS on a Thermo Dionex Ultimate 3000 LC-PDA system coupled to a Bruker Maxis impact QTOF system in an ESI positive mode and eluted with a gradient of H_2_O with 0.1% formic acid and CH_3_CN with a gradient method as follows: 10% CH_3_CN/H_2_O for 2 min, 10% CH_3_CN/H_2_O to 45% in 8 min, held at 45% CH_3_CN/H_2_O for 2 min, 45% CH_3_CN/H_2_O to 99% in 4 min, held at 99% CH_3_CN/H_2_O for 1 min, then 99% CH_3_CN/H_2_O to 10% CH_3_CN/H_2_O in 1 min, and finally held at 10% CH_3_CN/H_2_O for 2 min with the flow rate of 0.6 mL/min at room temperature. The UV chromatogram was measured at 210, 230, 280, 360 nm by photodiode array detection. Data-dependent (automated) MS/MS spectra were collected during the same run. The raw data of MS/MS spectra from the all fractions were converted to mzXML format using the ProteoWizard tool MSConvertGUI, and the processed files were uploaded to the GNPS website (http://gnps.ucsd.edu) to generate a molecular network that was visualized using Cytoscape 3.8 software (Weblinks S1 and S2). A molecular network was created using the online workflow on the GNPS website (https://ccms-ucsd.github.io/GNPSDocumentation). The precursor ion mass tolerance was set to 1 Da and a MS/MS fragment ion tolerance of 0.5 Da. The spectra in the network were then searched against available GNPS spectrometric libraries. The library spectra were filtered in the same manner as the input data. All matches kept between network spectra and library spectra were required to have a score above 0.7 and at least 4 matched peaks [33].

### 3.7. Cell Culture

The human colorectal cancer cell line, HCT116, was purchased from American Type Culture Collection (ATCC; Bethesda, MD, USA). Cells were maintained in DMEM medium, supplemented with 1% l-glutamine, 10% fetal bovine serum (FBS), 1% sodium pyruvate and 1% PenStrep (penicillin + streptomycin) (Biological Industries, Beit Haemek, Israel). Cells were grown in a humidified incubator at 37 °C with 5% CO_2_ in air, and served twice a week with fresh medium.

### 3.8. XTT Cell Proliferation Assay

Evaluation of the effect of each crude organic extract and fractions A-H, as well as subfractions C1-C6 and C3–1–C3–12 on cell viability was performed using the standard XTT assay and an established protocol [44]. In brief, HCT116 cells were seeded in 96-well plates (10^4^ cell/well) and 24 h later were treated for a period of 24 h with two doses from the crude extract and fractions A-H; 200 and 400 μg/mL, and with 4 doses for each subfraction; 15, 25, 50, and 100 μg/mL. Medium and DMSO were added to control wells. For sub-fraction C3–5, the XTT assay was additionally conducted using 30 μg/mL for 24 h. Following treatment, cell viability was determined by the XTT assay (Biological Industries, Beit Haemek, Israel) according to the manufacturer’s instructions using a plate reader (version, BioTek, Winooski, VT, USA). Experiments were repeated 3 times. Data were presented as the average proliferation percentage of the respective control.

### 3.9. Cell Cycle Analysis

A cell cycle evaluation experiment was carried out as described previously [44]. Briefly, 10^6^ cells were treated with 30 μg/mL of C3 or C3–5 for 24 h. At the end of treatment time, cells were trypsinized, harvested and centrifuged at 2000 rpm for 5 min at 4 ℃. Cells were washed with cold PBS and fixed with 70% EtOH for 1 h at −20 ℃. Cells were incubated with 0.1% NP-40 on ice for 5 min, followed by 30 min of incubation on ice with 100 μg/mL RNase (Sigma-Aldrich, St. Louis, MO, USA). Finally, 50 μg/mL propidium iodide (PI) was added to cells for 20 min. Cell cycle analysis was carried out by flow cytometry using a FACSCantoII with FACSDiva software (Becton Dickenson, San Jose, CA, USA); 10^4^ cells were counted for each the control and the treatment groups.

### 3.10. Annexin-V/PI Double Staining

Apoptotic cell death was evaluated and quantified using an Annexin-V FITC and PI double staining kit (Mebcyto^®^ Apoptosis Kit, MBL, Nagoya, Japan) according to the manufacturer’s instructions. In brief, 2 × 10^5^ HCT116 cells were seeded in 25 cm^2^ flasks. The next day, cells were treated with 30 μg/mL of C3 or **1** for 24 h. Both adherent and floating cells were collected in order to detect early and late apoptotic cells. Treated and untreated cells (control) were harvested by trypsinization, washed and suspended in ice-cold PBS. The washed cell pellets were re-suspended in an ice-cold binding buffer containing FITC-conjugated Annexin-V and PI. Samples were incubated at room temperature for 15 min in the dark before analysis by FACS, managed with FACSDiva software. The Annexin V-FITC-negative/PI negative, which are the normal healthy cells population are represented by quadrants Q3. Annexin V-FITC-positive/PI negative cells, which are defined as early apoptotic cells (Q4), whereas the Annexin V-FITC-positive/PI positive are the cells found in late apoptosis (Q2). The Annexin V-FITC-negative/PI-positive cells (Q1) include the necrotic cells. The percentage distributions of normal, early apoptotic, late apoptotic, and necrotic cells were calculated using FACSDiva software (Becton Dickenson, San Jose, CA, USA).

## 4. Conclusions

Cyanobacteria are vastly abundant organisms in various ecological niches, and marine filamentous cyanobacteria are a subset known to produce a treasure trove of natural products. Until challenges are overcome for using molecular biology tools to predict and realize the potential chemical arsenal of filamentous cyanobacteria via their biosynthetic gene clusters, a more complete chemical diversity of these organisms can be studied using large environmental collections. The chemical space of extracts produced from these assemblages is largely affected by external factors, such as the associated microbial consortia and environmental conditions, and thus increases the complexity of studying assemblages for new molecule discovery. Metabolomics-based approaches can be used to unravel the chemical potential of such complex samples, and minimize the rediscovery of previously reported compounds. The South China Sea harbors largely untapped filamentous cyanobacteria biodiversity that may be investigated to yield new pharmaceutical lead molecules. In this study, the investigation of a cf. *Neolyngbya* sp. cyanobacterium that was collected near Wenchang, Hainan, China led to the discovery of wenchangamide A (**1**) and characterization of its new chemical scaffold. Compound **1** was found to be a fast-acting and concentration-dependent inducer of apoptosis in HCT116 human colon cancer cells in vitro. Further untargeted LC-MS/MS-based metabolomics suggested the occurrence of an additional analogue, wenchangamide B, for which a structure has been proposed with high confidence. Bioassay results from the fraction containing this related molecule also showed in vitro apoptotic activity using HCT116 cells, suggesting that the core polypeptide-derived scaffold may be a pharmacophore and that the length of the polyketide chain could be tailoring molecules of this class for variable potency or solubility. The further expansion of this chemical class and structure–activity relationship should be evaluated for natural products anticancer drug discovery and development.

## Figures and Tables

**Table 1:** ^1^H and ^13^C NMR Spectroscopic Data for **1** in DMSO-*d*_6_ *^a,b^*.

Moiety	Position	*δ*_C_	Type	*δ*_H_, Mult (*J* in Hz)	Moiety	Position	*δ*_C_	Type	*δ*_H_, Mult (*J* in Hz)
AMP	1	62.7	CH_2_	3.35, m *^c^*	*N*-Me-Ile	33	169.4	C	
	2	52.4	CH	3.91, ddd (9.4, 5.1, 4.9)		34	59.9	CH	4.67, d (10.7)
	3	35.6	CH_2_	2.77, dd (14.0, 5.1); 2.53, dd (14.0, 4.9)		35	32.6	CH	1.9, m *^c^*
	4	130.9	C			36	25.5	CH_2_	1.35, m *^c^*; 0.99, m
	5, 9	130.0	CH	7.04, d (8.6)		37	11.3	CH_3_	0.81, m *^c^*
	6, 8	113.5	CH	6.79, d (8.6)		38	14.6	CH_3_	0.68, d (6.7)
	7	157.6	C			34-*N*-Me	30.5	CH_3_	2.94, s
	7-*O*-Me	55.0 *^c^*	CH_3_	3.71, s					
	2-NH			7.27, m*^c^*	Leu-2	39	173.0	C	
						40	47.4	CH	4.72, m
*N*-Me-Gln	10	169.2	C			41	40.5	CH_2_	1.46, m*^c^*; 1.35, m*^c^*
	11	56.0	CH	4.84, dd (5.1, 10.4) *^c^*		42	24.3	CH	1.61, m *^c^*
	12	23.8	CH_2_	1.97, m; 1.61, m *^c^*		43	23.1	CH_3_	0.87, d (6.6) ^*c*^
	13	31.3	CH_2_	1.90, m *^c^*; 1.83, m *^c^*		44	21.5	CH_3_	0.87, d (6.6) ^*c*^
	14	173.7	C			40-NH			8.15, d (7.8)
	14-NH_2_			7.28, m*^c^*; 6.71, br s					
	11-*N*-Me	29.8	CH_3_	2.42, s	FA	45	171.0	C	
						46	43.8	CH_2_	2.22, dd (14.0, 5.4)
*N*-Me-Phe	15	170.3	C						2.13, dd (14.0, 7.4)
	16	54.0	CH	5.62, dd (10.0, 5.9)		47	65.7	CH	3.87, m
	17	34.6	CH_2_	2.99 m; 2.94 m		48	45.2	CH_2_	1.23, m; 0.92, m *^c^*
	18	137.3	C			49	25.6	CH	1.76, m
	19, 23	129.5	CH	7.26, m		50	20.3	CH_3_	0.82, d (6.7)
	20, 22	128.0	CH	7.23, m		51	45.3	CH_2_	1.35, m *^c^*; 0.92, m *^c^*
	21	126.3	CH	7.17, t (6.7)		52	64.9	CH	3.55, m
	16-*N*-Me	30.1	CH_3_	2.89, s		53	46.7	CH_2_	1.26, m *^c^*; 1.04, m
						54	25.3	CH	1.83, m *^c^*
Leu-1	24	171.4	C			55	19.2	CH_3_	0.81, d (6.6) *^c^*
	25	46.9	CH	4.49, m		56	45.7	CH_2_	1.25 m *^c^*; 1.02 m *^c^*
	26	39.7*^c^*	CH_2_	0.95, m; 0.73, m		57	67.0	CH	3.46, m *^c^*
	27	23.7	CH	1.12, m		58	40.4	CH_2_	1.46, m *^c^*; 1.26, m *^c^*
	28	23.0	CH_3_	0.70, d (6.7)		59	18.6	CH_2_	1.35, m *^c^*; 1.26, m *^c^*
	29	21.7	CH_3_	0.69, d (6.7)		60	14.2	CH_2_	0.85, t (6.8)
	25-NH			8.04, d (7.9)		47-OH			4.53, m
						52-OH			4.11, br s
Ser	30	170.0	C			57-OH			4.17, br s
	31	55.0 *^c^*	CH	4.23, dt (7.8, 5.7)					
	32	61.6	CH_2_	3.43, m *^c^*					
	31-NH			7.53, d (7.8)					
	32-OH			4.84, m *^c^*					

*^a^* Data recorded at 298 K, 600 MHz (^1^H) and 150 MHz (^13^C). *^b^* Assignments supported by 2D NMR. *^c^* Signal partially overlapped.

## Data Availability

The datasets generated for this study can be found in the online supplementary materials. Metabolomics data are archived on the GNPS platform and can be found in the following links: https://gnps.ucsd.edu/ProteoSAFe/status.jsp?task=f62b23918fb24bca9f4a234f3555df50; https://gnps.ucsd.edu/ProteoSAFe/status.jsp?task=0e36af9bc15d4d6c901292d5be8ff32b.

## Supplementary Materials

The following are available online at https://www.mdpi.com/article/10.3390/md19070397/s1: ^1^H, ^13^C, DEPT135, COSY, TOCSY, HSQC-TOCSY, HSQC, HMBC, and ROESY NMR, UV, and IR for compound **1**. Tabulated NMR data for **1** in pyridine-*d*_5_. MS/MS spectra for compound **1** and the proposed analogue, wenchangamide B. Chiral HPLC chromatograms of hydrolysates of **1** and authentic standards. In vitro bioassay data. Weblinks for the GNPS metabolomic data used to produce Figure 3 and Figure 9. 16S rRNA gene V3–V4 amplicon used to prepare Figure 2C.

## References

[1] Salvador-Reyes L.A., Luesch H. (2015). Biological targets and mechanisms of action of natural products from marine cyanobacteria. Nat. Prod. Rep..

[2] Demay J., Bernard C., Reinhardt A., Marie B. (2019). Natural products from cyanobacteria: Focus on beneficial activities. Mar. Drugs.

[3] Tan L.T., Phyo M.Y. (2020). Marine cyanobacteria: A source of lead compounds and their clinically-relevant molecular targets. Molecules.

[4] Mayer A.M.S., Guerrero A.J., Rodríguez A.D., Taglialatela-Scafati O., Nakamura F., Fusetani N. (2020). Marine pharmacology in 2014–2015: Marine compounds with antibacterial, antidiabetic, antifungal, anti-inflammatory, antiprotozoal, antituberculosis, antiviral, and anthelmintic activities; affecting the immune and nervous systems, and other miscellaneous mechanisms of action. Mar. Drugs.

[5] Jang M.-H., Ha K., Lucas M.C., Joo G.-J., Takamura N. (2004). Changes in microcystin production by *Microcystis aeruginosa* exposed to phytoplanktivorous and omnivorous fish. Aquat. Toxicol..

[6] Jiang X., Gao H., Zhang L., Liang H., Zhu X. (2016). Rapid evolution of tolerance to toxic *Microcystis* in two cladoceran grazers. Sci. Rep..

[7] Leao T., Castelão G., Korobeynikov A., Monroe E.A., Podell S., Glukhov E., Allen E.E., Gerwick W.H., Gerwick L. (2017). Comparative genomics uncovers the prolific and distinctive metabolic potential of the cyanobacterial genus *Moorea*. Proc. Natl. Acad. Sci. USA.

[8] Moss N.A., Leao T., Glukhov E., Gerwick L., Gerwick W.H., Moore B.S. (2018). Collection, Culturing, and Genome Analyses of Tropical Marine Filamentous Benthic Cyanobacteria. Methods in Enzymology.

[9] Crnkovic C.M., May D.S., Orjala J. (2018). The impact of culture conditions on growth and metabolomic profiles of freshwater cyanobacteria. J. Appl. Phycol..

[10] Pye C.R., Bertin M.J., Lokey R.S., Gerwick W.H., Linington R.G. (2017). Retrospective analysis of natural products provides insights for future discovery trends. Proc. Natl. Acad. Sci. USA.

[11] Hašler P., Dvořák P., Johansen J.R., Kitner M., Ondřej V., Poulíčková A. (2012). Morphological and molecular study of epipelic filamentous genera *Phormidium*, *Microcoleus* and *Geitlerinema* (Oscillatoriales, Cyanophyta/cyanobacteria). Fottea-Olomouc.

[12] Stoyanov P., Moten D., Mladenov R., Dzhambazov B., Teneva I. (2014). Phylogenetic relationships of some filamentous cyanoprokaryotic species. Evol. Bioinform..

[13] Nuryadi H., Sumimoto S., Teruya T., Suenaga K., Suda S. (2020). Characterization of macroscopic colony-forming filamentous cyanobacteria from Okinawan coasts as potential sources of bioactive compounds. Mar. Biotechnol..

[14] Anagnostidis K. (2001). Nomenclatural changes in cyanoprokaryotic order Oscillatoriales. Preslia Praha.

[15] Hoffmann L., Komárek J., Kaštovský J. (2005). System of cyanoprokaryotes (cyanobacteria) state in 2004. Arch. Hydrobiol. Suppl. Algol. Stud..

[16] Komárek J., Johansen J.R. (2003). Filamentous Cyanobacteria. Freshwater Algae of North America: Ecology and Classification.

[17] Komárek J., Johansen J.R. (2015). Filamentous Cyanobacteria. Freshwater Algae of North America: Ecology and Classification.

[18] Komárek J., Kaštovský J., Mareš J., Johansen J.R. (2014). Taxonomic classification of cyanoprokaryotes (cyanobacterial genera) 2014, using a polyphasic approach. Preslia.

[19] Engene N., Paul V.J., Byrum T., Gerwick W.H., Thor A., Ellisman M.H. (2013). Five chemically rich species of tropical marine cyanobacteria of the genus *Okeania* gen. nov. (Oscillatoriales, Cyanoprokaryota). J. Phycol..

[20] Tronholm A., Engene N. (2019). *Moorena gen. nov.*, a valid name for “*Moorea* Engene & *al.*” nom. inval. (*Oscillatoriaceae*, *Cyanobacteria*). Not. Algarum.

[21] Caires T.A., de Mattos Lyra G., Hentschke G.S., de Gusmão Pedrini A., Sant’Anna C.L., de Castro Nunes J.M. (2018). *Neolyngbya* gen. nov. (Cyanobacteria, Oscillatoriaceae): A new filamentous benthic marine taxon widely distributed along the Brazilian coast. Mol. Phylogenet. Evol..

[22] Caires T.A., da Silva A.M.S., Vasconcelos V.M., Affe H.M.J., de Souza Neta L.C., Boness H.V.M., Sant’Anna C.L., Nunes J.M.C. (2018). Biotechnological potential of *Neolyngbya* (Cyanobacteria), a new marine benthic filamentous genus from Brazil. Algal Res..

[23] Lydon C.A., Mathivathanan L., Sanchez J., dos Santos L.A.H., Sauvage T., Gunasekera S.P., Paul V.J., Berry J.P. (2020). Eudesmacarbonate, a eudesmane-type sesquiterpene from a marine filamentous cyanobacterial mat (Oscillatoriales) in the Florida Keys. J. Nat. Prod..

[24] Guan H., Wang S. (2009). Algae. Chinese Marine Materia Medica.

[25] Titlyanov E.A., Titlyanova T.V., Li X., Huang H., Titlyanov E.A., Titlyanova T.V., Li X., Huang H. (2017). Chapter 2—Marine Plants of Coral Reefs. Coral Reef Marine Plants of Hainan Island.

[26] Sun W., Wu W., Liu X., Zaleta-Pinet D.A., Clark B.R. (2019). Bioactive compounds isolated from marine-derived microbes in China: 2009-2018. Mar. Drugs.

[27] Demarque D.P., Dusi R.G., de Sousa F.D.M., Grossi S.M., Silvério M.R.S., Lopes N.P., Espindola L.S. (2020). Mass spectrometry-based metabolomics approach in the isolation of bioactive natural products. Sci. Rep..

[28] Luzzatto-Knaan T., Garg N., Wang M., Glukhov E., Peng Y., Ackermann G., Amir A., Duggan B.M., Ryazanov S., Gerwick L. (2017). Digitizing mass spectrometry data to explore the chemical diversity and distribution of marine cyanobacteria and algae. eLife.

[29] Luzzatto-Knaan T., Melnik A.V., Dorrestein P.C. (2015). Mass spectrometry tools and workflows for revealing microbial chemistry. Analyst.

[30] Yang J.Y., Sanchez L.M., Rath C.M., Liu X., Boudreau P.D., Bruns N., Glukhov E., Wodtke A., de Felicio R., Fenner A. (2013). Molecular networking as a dereplication strategy. J. Nat. Prod..

[31] Olivon F., Allard P.-M., Koval A., Righi D., Genta-Jouve G., Neyts J., Apel C., Pannecouque C., Nothias L.-F., Cachet X. (2017). Bioactive natural products prioritization using massive multi-informational molecular networks. ACS Chem. Biol..

[32] Nothias L.F., Nothias-Esposito M., da Silva R., Wang M., Protsyuk I., Zhang Z., Sarvepalli A., Leyssen P., Touboul D., Costa J. (2018). Bioactivity-based molecular networking for the discovery of drug leads in natural product bioassay-guided fractionation. J. Nat. Prod..

[33] Wang M., Carver J.J., Phelan V.V., Sanchez L.M., Garg N., Peng Y., Nguyen D.D., Watrous J., Kapono C.A., Luzzatto-Knaan T. (2016). Sharing and community curation of mass spectrometry data with Global Natural Products Social Molecular Networking. Nat. Biotechnol..

[34] Wolfender J.L., Litaudon M., Touboul D., Queiroz E.F. (2019). Innovative omics-based approaches for prioritisation and targeted isolation of natural products—New strategies for drug discovery. Nat. Prod. Rep..

[35] Fox Ramos A.E., Evanno L., Poupon E., Champy P., Beniddir M.A. (2019). Natural products targeting strategies involving molecular networking: Different manners, one goal. Nat. Prod. Rep..

[36] Ernst M., Kang K.B., Caraballo-Rodríguez A.M., Nothias L.F., Wandy J., Wang M., Rogers S., Medema M.H., Dorrestein P.C., van der Hooft J.J.J. (2019). MolNetEnhancer: Enhanced molecular networks by integrating metabolome mining and annotation tools. Metabolites.

[37] Cornet L., Bertrand A.R., Hanikenne M., Javaux E.J., Wilmotte A., Baurain D. (2018). Metagenomic assembly of new (sub)polar cyanobacteria and their associated microbiome from non-axenic cultures. Microb. Genom..

[38] Gris B., Treu L., Zampieri R.M., Caldara F., Romualdi C., Campanaro S., La Rocca N. (2020). Microbiota of the therapeutic Euganean thermal muds with a focus on the main cyanobacteria species. Microorganisms.

[39] Gogineni V., Hamann M.T. (2018). Marine natural product peptides with therapeutic potential: Chemistry, biosynthesis, and pharmacology. Biochim. Biophys. Acta Gen. Subj..

[40] Gross H., König G.M. (2006). Terpenoids from marine organisms: Unique structures and their pharmacological potential. Phytochem. Rev..

[41] Sorokina M., Steinbeck C. (2020). Review on natural products databases: Where to find data in 2020. J. Cheminform..

[42] Wang H., Fewer D.P., Holm L., Rouhiainen L., Sivonen K. (2014). Atlas of nonribosomal peptide and polyketide biosynthetic pathways reveals common occurrence of nonmodular enzymes. Proc. Natl. Acad. Sci. USA.

[43] Naman C.B., Rattan R., Nikoulina S.E., Lee J., Miller B.W., Moss N.A., Armstrong L., Boudreau P.D., Debonsi H.M., Valeriote F.A. (2017). Integrating molecular networking and biological assays to target the isolation of a cytotoxic cyclic octapeptide, samoamide A, from an American Samoan marine cyanobacterium. J. Nat. Prod..

[44] Bar-Shalom R., Bergman M., Grossman S., Azzam N., Sharvit L., Fares F. (2019). *Inula viscosa* extract inhibits growth of colorectal cancer cells *in vitro* and *in vivo* through induction of apoptosis. Front. Oncol..

[45] Zheng Q., Hirose Y., Yoshimi N., Murakami A., Koshimizu K., Ohigashi H., Sakata K., Matsumoto Y., Sayama Y., Mori H. (2002). Further investigation of the modifying effect of various chemopreventive agents on apoptosis and cell proliferation in human colon cancer cells. J. Cancer Res. Clin. Oncol..

[46] Sumimoto S., Kobayashi M., Sato R., Shinomiya S., Iwasaki A., Suda S., Teruya T., Inuzuka T., Ohno O., Suenaga K. (2019). Minnamide A, a linear lipopeptide from the marine cyanobacterium *Okeania hirsuta*. Org. Lett..

[47] Liang X., Matthew S., Chen Q.Y., Kwan J.C., Paul V.J., Luesch H. (2019). Discovery and total synthesis of doscadenamide A: A quorum sensing signaling molecule from a marine cyanobacterium. Org. Lett..

[48] Leber C.A., Naman C.B., Keller L., Almaliti J., Caro-Diaz E.J.E., Glukhov E., Joseph V., Sajeevan T.P., Reyes A.J., Biggs J.S. (2020). Applying a chemogeographic strategy for natural product discovery from the marine cyanobacterium *Moorena bouillonii*. Mar. Drugs.

[49] Liang X., Chen Q.-Y., Seabra G.M., Matthew S., Kwan J.C., Li C., Paul V.J., Luesch H. (2021). Bifunctional doscadenamides activate quorum sensing in gram-negative bacteria and synergize with TRAIL to induce apoptosis in cancer cells. J. Nat. Prod..

[50] Ondov B.D., Bergman N.H., Phillippy A.M. (2011). Interactive metagenomic visualization in a Web browser. BMC Bioinform..

[51] Pruesse E., Peplies J., Glöckner F.O. (2012). SINA: Accurate high-throughput multiple sequence alignment of ribosomal RNA genes. Bioinformatics.

