# Supplementary material for: Metabolomic Characterization of a cf. Neolyngbya Cyanobacterium from the South China Sea Reveals Wenchangamide A, a Lipopeptide with In Vitro Apoptotic Potential in Colon Cancer Cells
Source: Mar Drugs. 2021 Jul 16;19(7):397. doi: 10.3390/md19070397 (PMC8307421; doi:10.3390/md19070397)
Supplement: Supplementary file 1 [file marinedrugs-19-00397-s001.zip › marinedrugs-1227033-supplementary.pdf]

# Supplementary Materials

# Metabolomic Characterization of a cf. *Neolyngbya* Cyanobacterium from the South China Sea Reveals Wenchangamide A, a Lipopeptide with In Vitro Apoptotic Potential in Colon Cancer Cells

**Lijian Ding [1,2,†], Rinat Bar-Shalom [3,†], Dikla Aharonovich [2], Naoaki Kurisawa [4], Gaurav Patial [1], Shuang Li [1], Shan He [1], Xiaojun Yan [1], Arihiro Iwasaki [4,5], Kiyotake Suenaga [4,5], Chengchong Zhu [5], Haixi Luo [5], Fuli Tian [5], Fuad Fares [3], C. Benjamin Naman [1,5,*] and Tal Luzzatto-Knaan [2,*]**

[1] Li Dak Sum Yip Yio Chin Kenneth Li Marine Biopharmaceutical Research Center, Department of Marine Pharmacy, College of Food and Pharmaceutical Sciences, Ningbo University, Ningbo 315800, China;
[2] Department of Marine Biology, Leon H. Charney School of Marine Sciences, University of Haifa, Haifa 3498838, Israel;
[3] Department of Human Biology, Faculty of Life Sciences, University of Haifa, Haifa 31905, Israel;
[4] Department of Chemistry, Keio University, 3-14-1, Hiyoshi, Kohoku-ku, Yokohama, Kanagawa 223-8522, Japan;
[5] Key Laboratory of Medicinal and Edible Plant Resources of Hainan Province, Hainan Vocational University of Science and Technology, Haikou 571126, China;
* Correspondence: bnaman@nbu.edu.cn (C.B.N.); tluzzatto@univ.haifa.ac.il (T.L.-K.)
† These authors contributed equally to the work.

**Table of Contents**

**Figure S1**. $^1H$ NMR spectrum of **1** in DMSO-$d_6$. ..... 3

**Figure S2**. $^{13}C$ NMR spectrum of **1** in DMSO-$d_6$. ..... 3

**Figure S3**. $^{13}C$ DEPT135 NMR spectrum of **1** in DMSO-$d_6$. ..... 4

**Figure S4**. $^1H$-$^1H$ COSY NMR spectrum of **1** in DMSO-$d_6$. ..... 4

**Figure S5**. $^1H$-$^1H$ TOCSY NMR spectrum of **1** in DMSO-$d_6$. ..... 5

**Figure S6**. $^1H$-$^{13}C$ HSQC-TOCSY NMR spectrum of **1** in DMSO-$d_6$. ..... 5

**Figure S7**. $^1H$-$^{13}C$ HSQC NMR spectrum of **1** in DMSO-$d_6$. ..... 6

**Figure S8**. $^1H$-$^{13}C$ HMBC NMR spectrum of **1** in DMSO-$d_6$. ..... 6

**Figure S9**. $^1H$-$^{13}C$ HMBC NMR spectrum of **1** in DMSO-$d_6$ (expanded ester carbonyl region). ..... 7

**Figure S10**. $^1H$-$^1H$ ROESY NMR spectrum of **1** in DMSO-$d_6$. ..... 7

**Table S1**. $^1H$ and $^{13}C$ NMR Spectroscopic Data for **1** in pyridine-$d_5$. ..... 8

**Figure S11**. UV spectrum of **1** in MeOH at 0.25 mg/mL. ..... 9

**Figure S12**. IR spectrum of **1** in KBr. ..... 9

**Figure S13**. HRESIMS/MS of **1**. ..... 10

**Figure S14**. HRESIMS/MS spectrum of **1** (expanded view *m/z* 600-1200). ..... 10

**Figure S15**. HRESIMS/MS spectrum of **1** (expanded view *m/z* 0-600). ..... 11

**Figure S16**. MS/MS spectra of wenchangamide A (**1**; *m/z* 1211) in C3-5 and wenchangamide B (proposed; *m/z* 1297) in C3-7. ..... 11

**Figure S17**. Chiral HPLC analysis of hydrosylates of **1** (Ser). ..... 12

**Figure S18**. Chiral HPLC analysis of hydrosylates of **1** (Leu). ..... 13

**Figure S19**. Chiral HPLC analysis of hydrosylates of **1** (*N*-Me-Ile). ..... 14

**Figure S20**. Chiral HPLC analysis of hydrosylates of **1** (*N*-Me-Glu). ..... 15

**Figure S21**. Chiral HPLC analysis of hydrosylates of **1** (*N*-Me-Phe). ..... 16

**Figure S22**. Chiral HPLC analysis of hydrosylates of **1** (AMP). ..... 17

**Figure S23**. Bioactivity data used to guide fractionation of cf. *Neolyngbya* sp. extract using HCT116 human colorectal cancer cell viability *in vitro*. ..... 18

**Figure S24**. The *in vitro* effect of C3, C3-5 (compound **1**), and C3-7 on HCT116 cells viability, following 24 h of treatment with escalating concentrations. ..... 19

**Figure S25**. The *in vitro* effect of C3-5 (compound **1**) on HCT116 cells viability, following 8 h of treatment with escalating concentrations. ..... 19

**Weblink S1**. GNPS metabolomics data used to prepare main text Figure 4. ..... 20

**Weblink S2**. GNPS metabolomics data used to prepare main text Figure 9. ..... 20

**Sequence S1**. 16S rRNA gene V3-V4 amplicon used to prepare main text Figure 3C. ..... 20

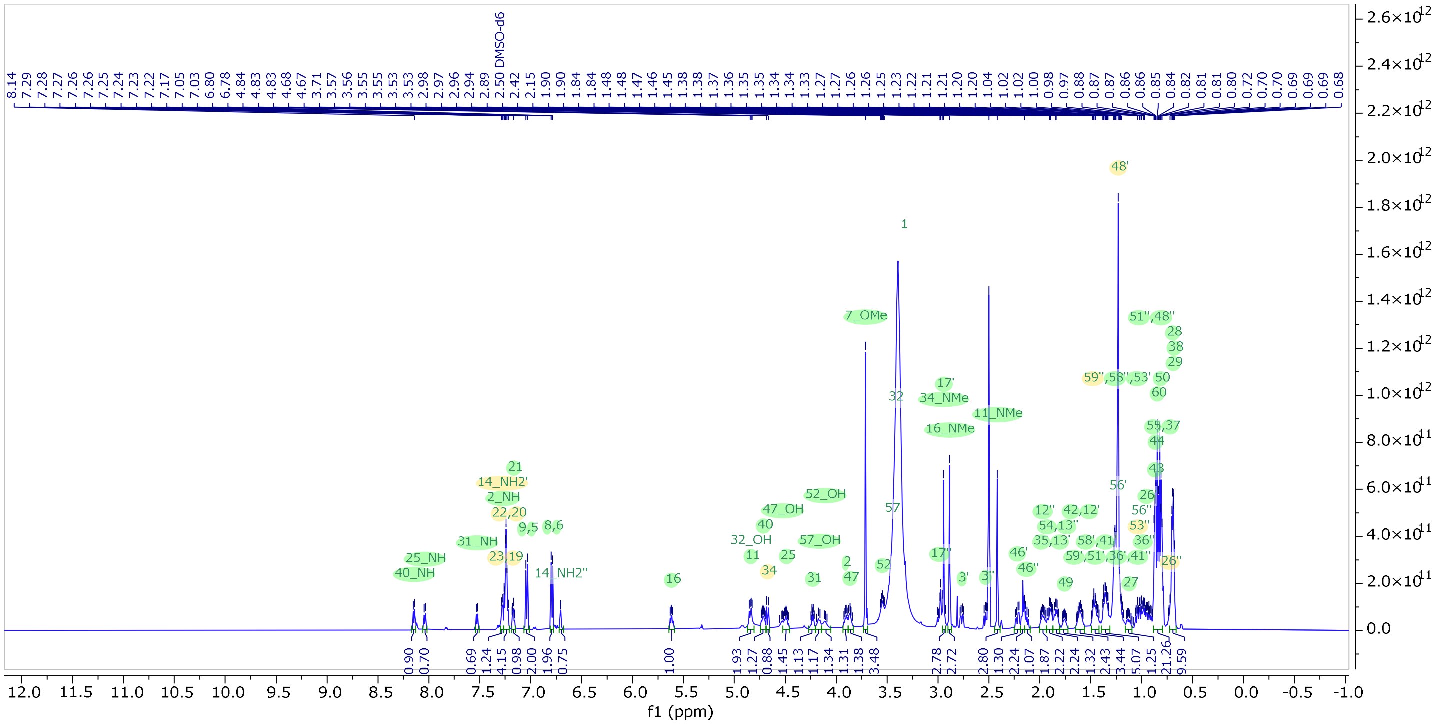


**Figure S1.** $^{1}$H NMR spectrum of **1** in DMSO-*d*6.

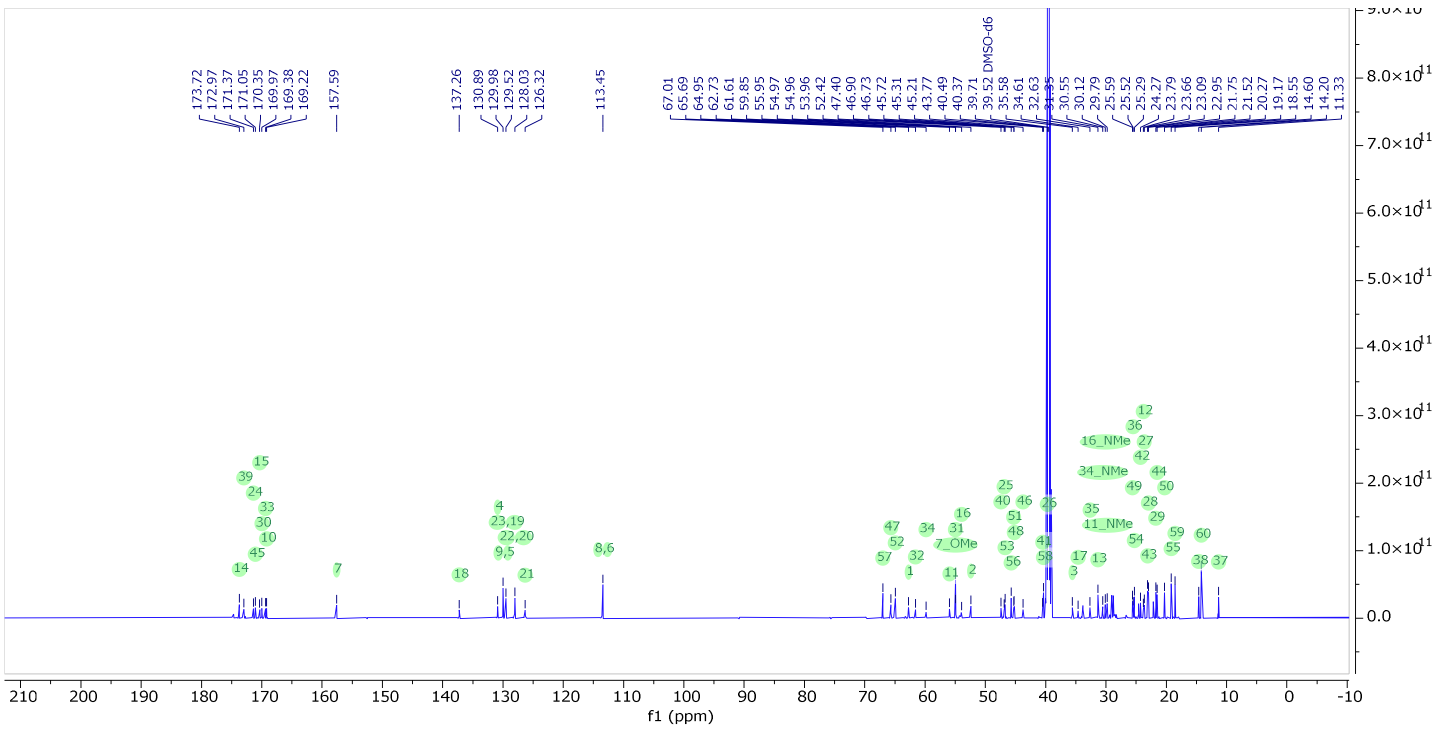


**Figure S2.** $^{13}$C NMR spectrum of **1** in DMSO-*d*6.

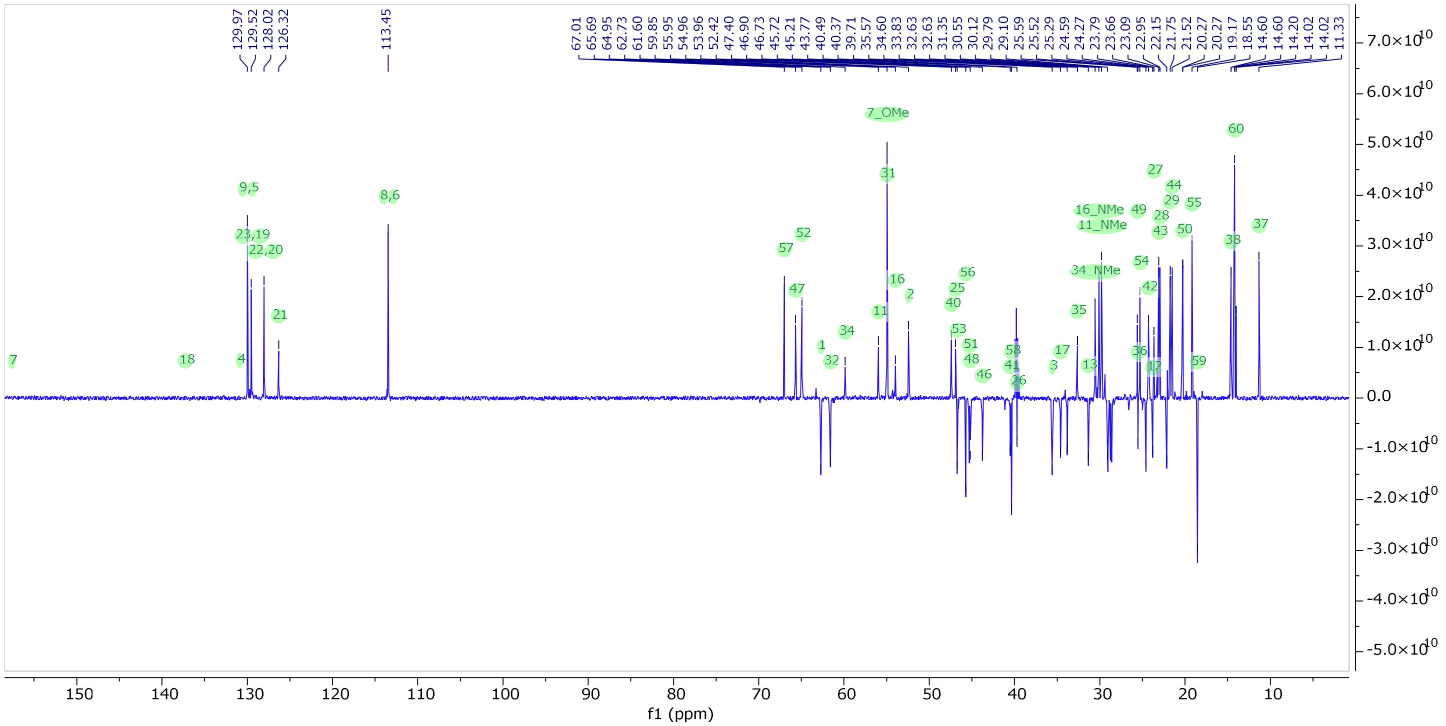


**Figure S3.** $^{13}$C DEPT135 NMR spectrum of **1** in DMSO-$d_6$.

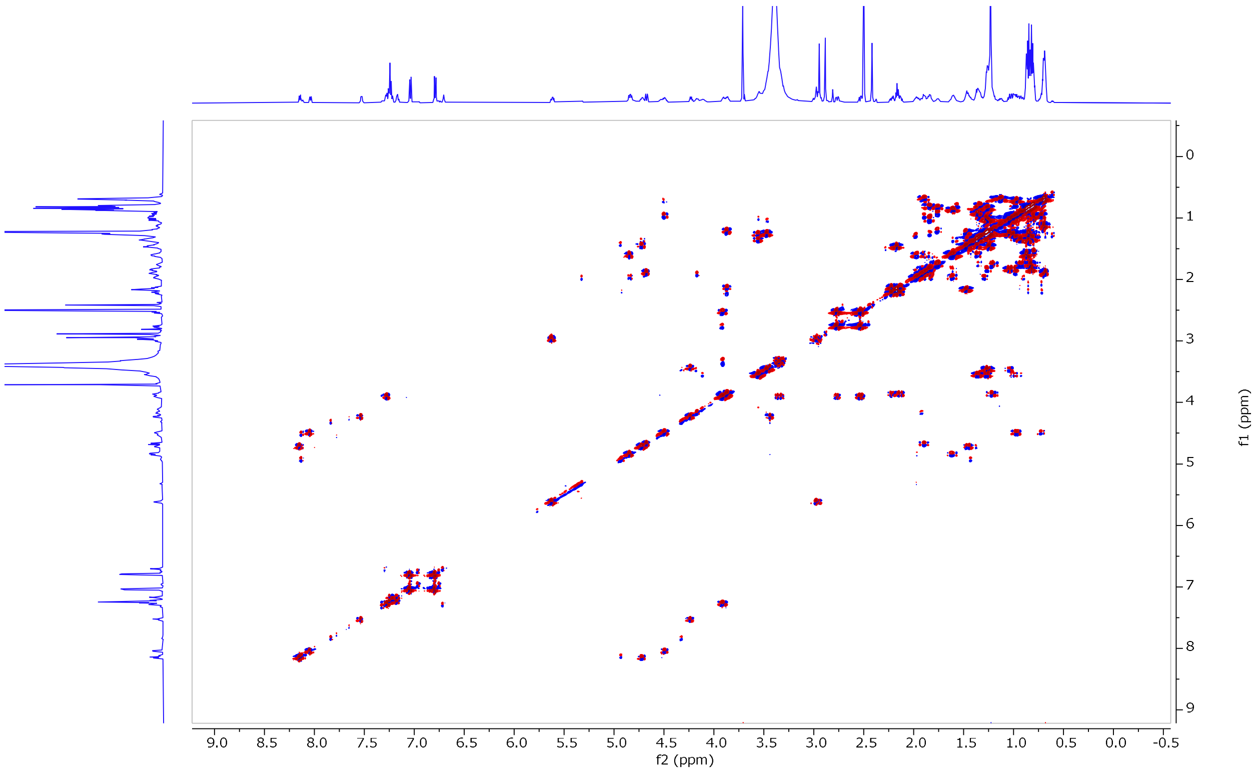


**Figure S4.** $^{1}$H-$^{1}$H COSY NMR spectrum of **1** in DMSO-$d_6$.

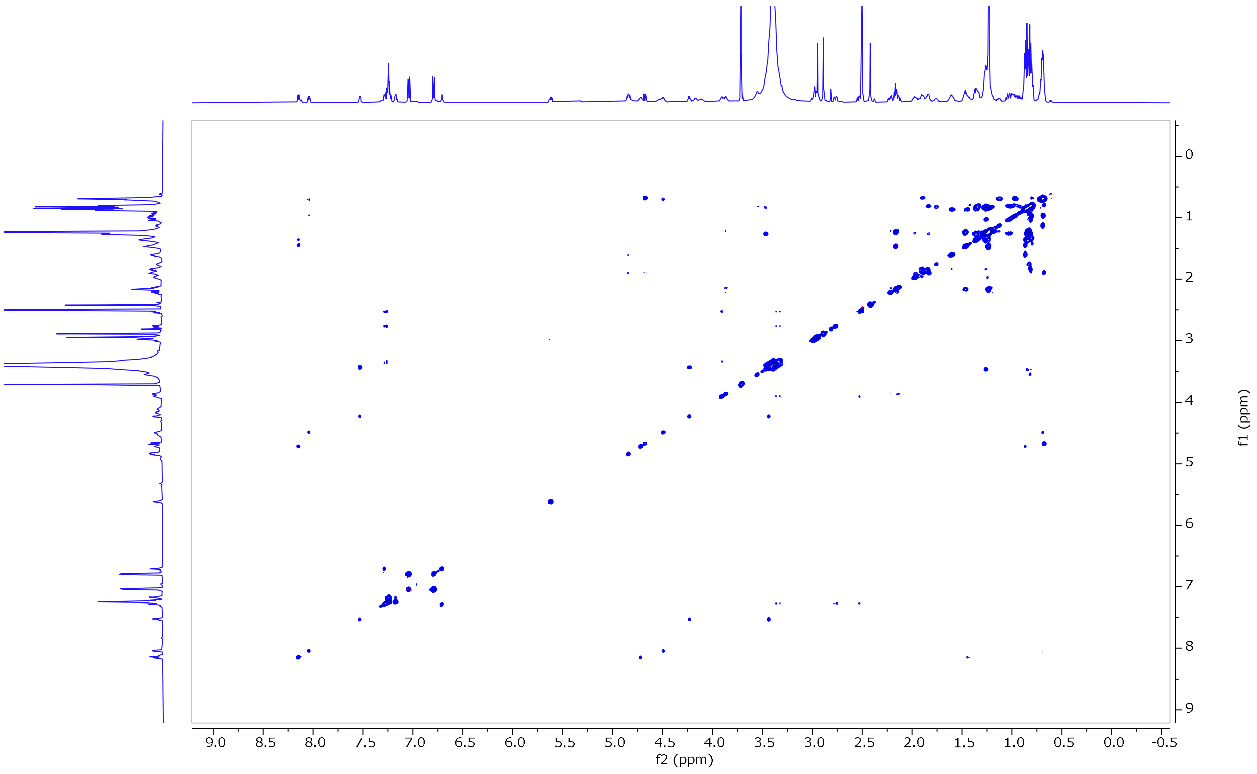


**Figure S5.** $^{1}H$-$^{1}H$ TOCSY NMR spectrum of **1** in DMSO-*d*$_6$.

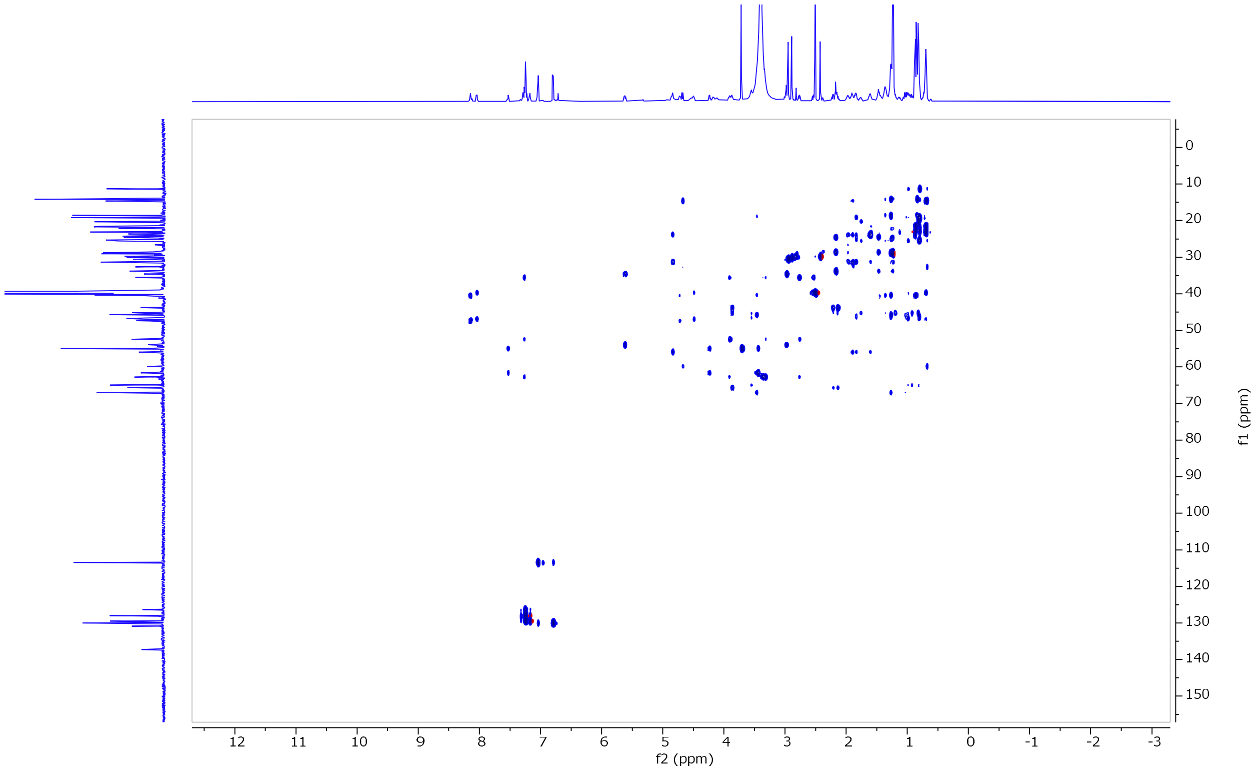


**Figure S6.** $^{1}H$-$^{13}C$ HSQC-TOCSY NMR spectrum of **1** in DMSO-*d*$_6$.

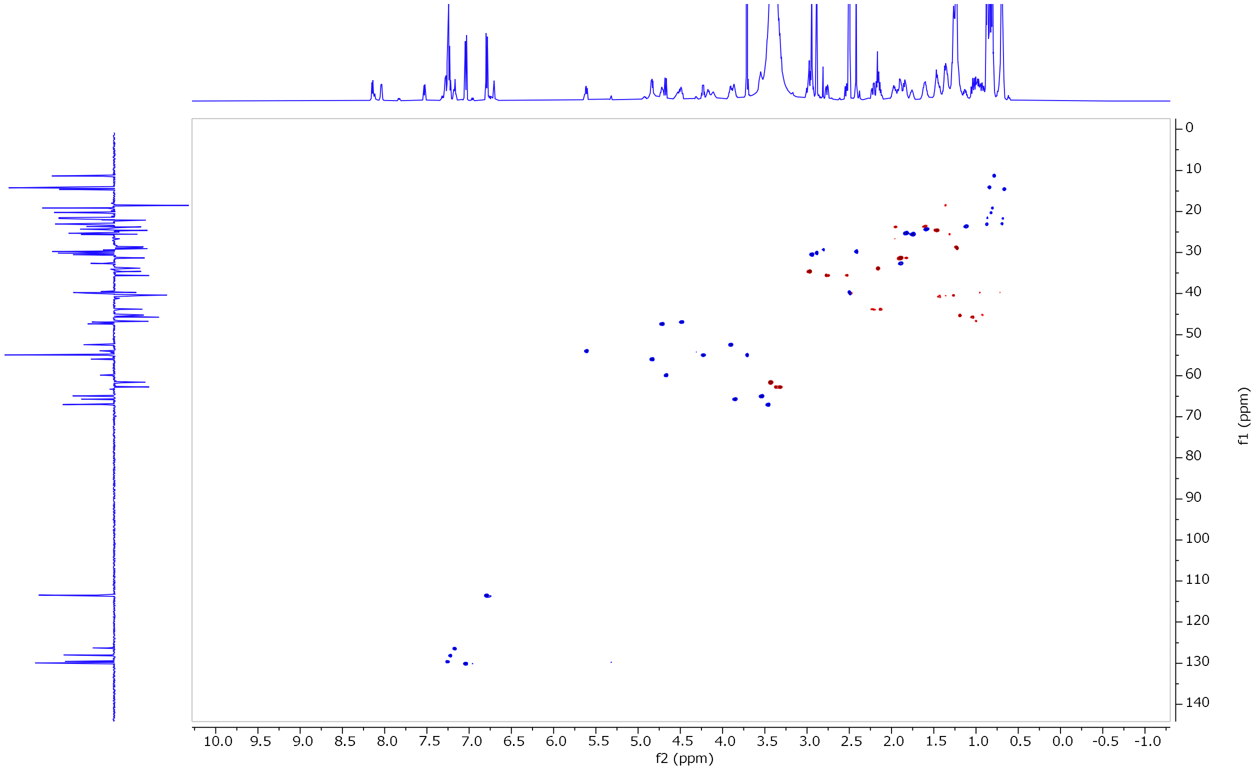


**Figure S7.** $^{1}H$-$^{13}C$ HSQC NMR spectrum of **1** in DMSO-*d*$_6$.

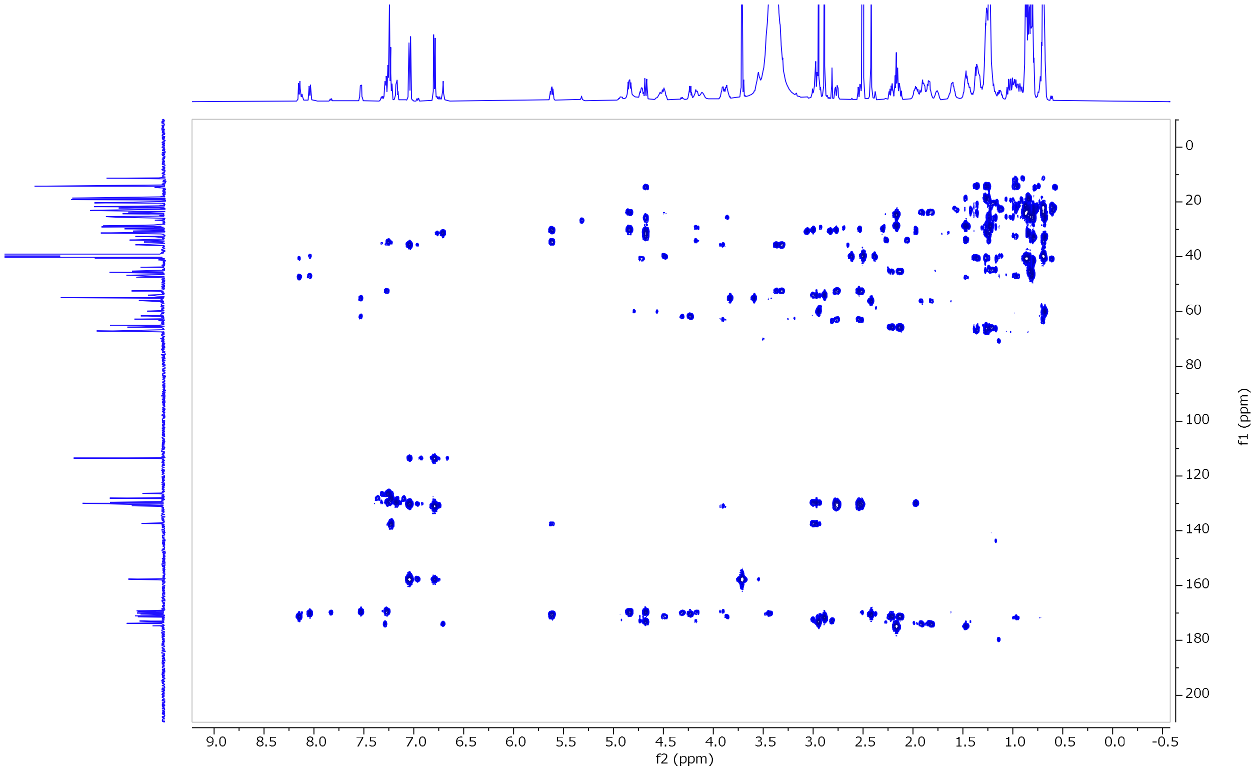


**Figure S8.** $^{1}H$-$^{13}C$ HMBC NMR spectrum of **1** in DMSO-*d*$_6$.

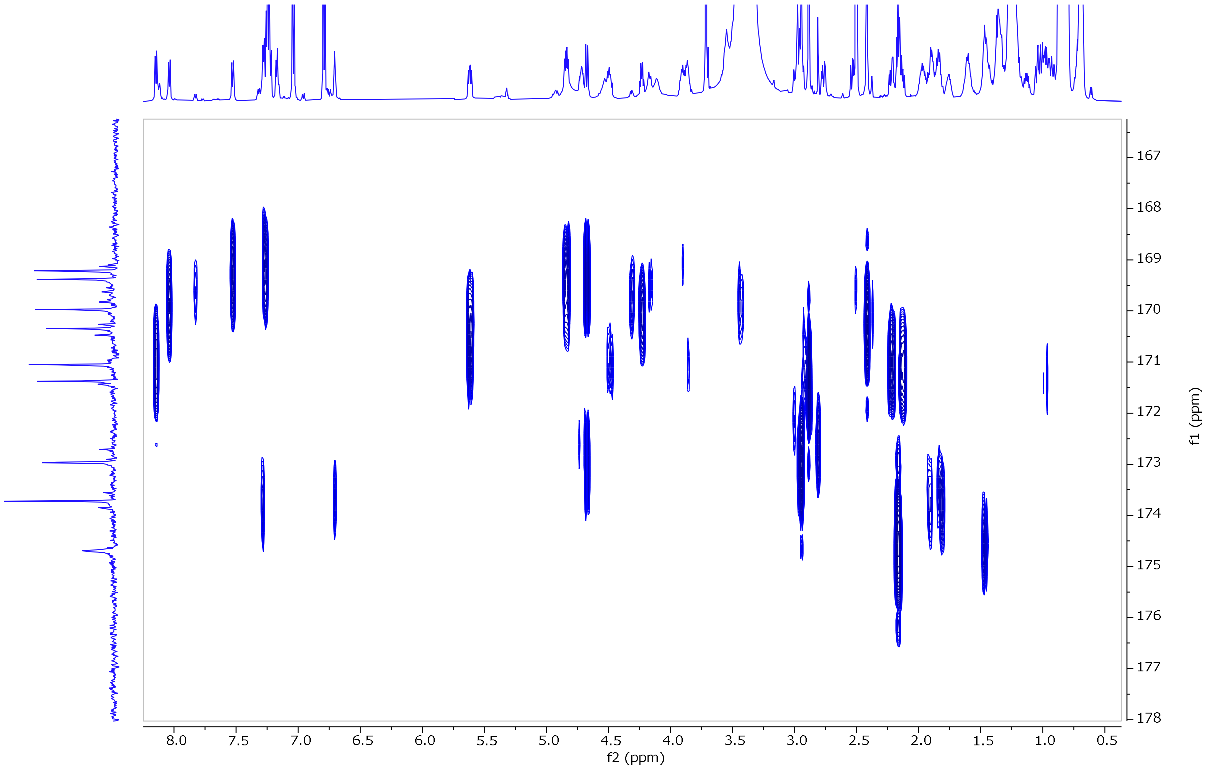


**Figure S9.** $^{1}H$-$^{13}C$ HMBC NMR spectrum of **1** in DMSO-$d_6$ (expanded ester carbonyl region).

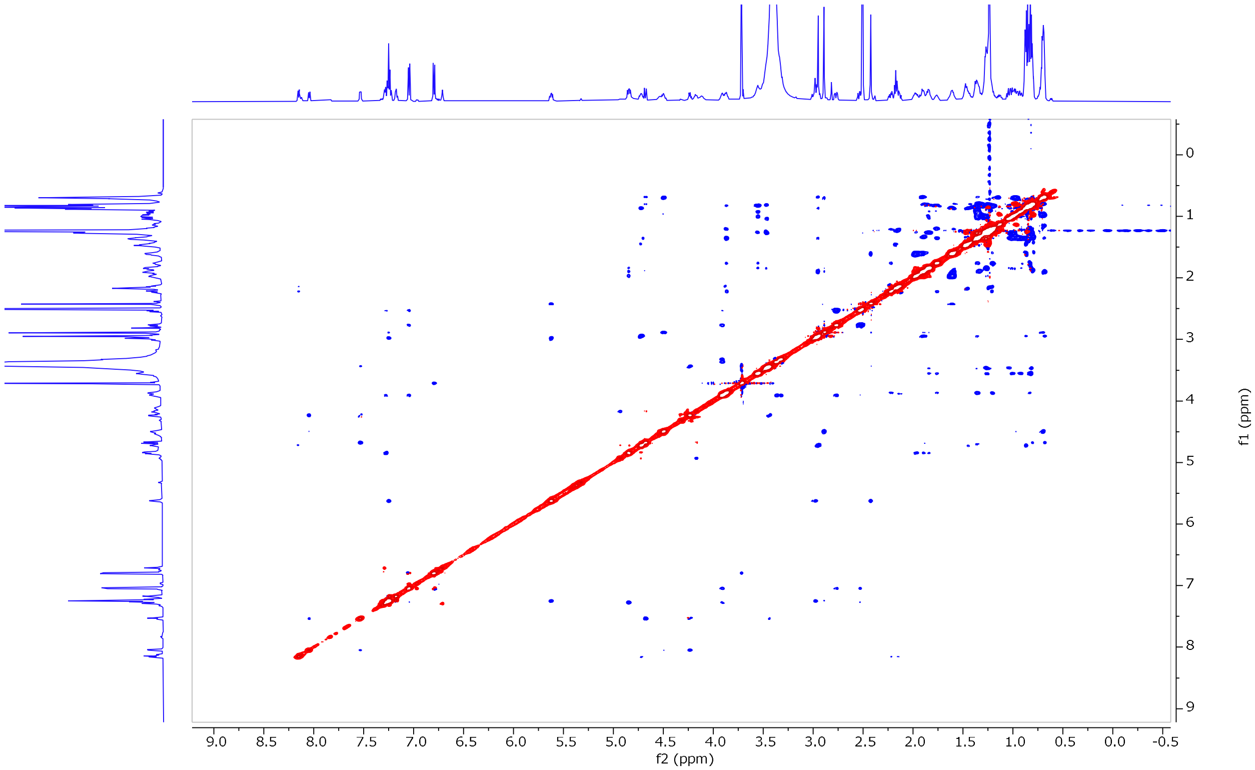


**Figure S10.** $^{1}H$-$^{1}H$ ROESY NMR spectrum of **1** in DMSO-$d_6$.

**Table S1.** $^{1}$H and $^{13}$C NMR Spectroscopic Data for **1** in pyridine-*d*$_5$.[a,b]

| Moiety | Position | $\delta_C$ | Type | $\delta_H$, mult (*J* in Hz) | Moiety | Position | $\delta_C$ | Type | $\delta_H$, mult (*J* in Hz) |
|---|---|---|---|---|---|---|---|---|---|
| AMP | 1 | 63.6 | $CH_2$ | 3.92, m | *N*-Me-Ile | 33 | 170.0 | C | |
| | 2 | 53.2 | CH | 4.65, m | | 34 | 61.2 | CH | 5.24 d (10.6) |
| | 3 | 36.7 | $CH_2$ | 2.77, dd (13.9, 5.8); 2.53, dd (13.9, 8.8) | | 35 | 32.7 | CH | 2.23, m |
| | 4 | 131.4 | C | | | 36 | 26.9 | $CH_2$ | 1.67, m; 1.01, m |
| | 5, 9 | 130.7 | CH | 7.04, d (8.5) | | 37 | 11.6 | $CH_3$ | 0.81, t (7.2) |
| | 6, 8 | 114.3 | CH | 6.79, d (8.5) | | 38 | 14.9 | $CH_3$ | 0.79, d (6.5) |
| | 7 | 158.7 | C | | | 34-*N*-Me | 31.3 | $CH_3$ | 3.26, s |
| | 7-*O*-Me | 55.3c | $CH_3$ | 3.71, s | | | | | |
| | 2-NH | | | 7.85, d (8.8) | Leu-2 | 39 | 171.5 | C | |
| | | | | | | 40 | 48.7 | CH | 5.22, m |
| *N*-Me-Gln | 10 | 170.8 | C | | | 41 | 41.4 | $CH_2$ | 1.81, m |
| | 11 | 57.3 | CH | 5.49, dd (11.7, 4.3) | | 42 | 25.2 | CH | 1.87, m |
| | 12 | 24.9 | $CH_2$ | 2.67, m; 2.51, m | | 43 | 23.0 | $CH_3$ | 0.87, d (6.3) |
| | 13 | 32.5 | $CH_2$ | 2.53, m; 2.33, m | | 44 | 22.4 | $CH_3$ | 0.94, d (6.1) |
| | 14 | 176.1 | C | | | 40-NH | | | 9.28, d (7.2) |
| | 14-$NH_2$ | | | 8.26, m; 7.52, m | | | | | |
| | 11-*N*-Me | 30.8 | $CH_3$ | 3.10, s | FA | 45 | 173.4 | C | |
| | | | | | | 46 | 44.5 | $CH_2$ | 2.81, dd (13.7, 3.6) |
| *N*-Me-Phe | 15 | 172.0 | C | | | | | | 2.76, dd (13.7, 8.3) |
| | 16 | 54.6 | CH | 6.15, dd (10.1, 6.1) | | 47 | 66.8 | CH | 4.63, m |
| | 17 | 35.6 | $CH_2$ | 3.29, dd (13.9, 6.1) | | 48 | 46.6 | $CH_2$ | 1.81, m; 1.67, m |
| | | | | 3.20, dd (13.9, 10.1) | | 49 | 26.8 | CH | 2.41, m |
| | 18 | 138.0 | C | | | 50 | 20.6 | $CH_3$ | 1.10, d (6.5) |
| | 19, 23 | 130.3 | CH | 7.37, d (7.2) | | 51 | 46.2 | $CH_2$ | 1.91, m; 1.29, m |
| | 20, 22 | 128.7 | CH | 7.27, dd (7.4, 7.2) | | 52 | 66.5 | CH | 4.13, m |
| | 21 | 126.9 | CH | 7.17, t (7.4) | | 53 | 46.9 | $CH_2$ | 1.78, m; 1.36, m |
| | 16-*N*-Me | 30.7 | $CH_3$ | 3.25, s | | 54 | 26.6 | CH | 2.57, m |
| | | | | | | 55 | 19.6 | $CH_3$ | 1.11, d (6.5) |
| Leu-1 | 24 | 172.7 | C | | | 56 | 48.0 | $CH_2$ | 1.76 m; 1.37 m |
| | 25 | 47.9 | CH | 5.03, m | | 57 | 68.5 | CH | 3.97, m |
| | 26 | 40.4 | $CH_2$ | 1.37, m; 1.07, m | | 58 | 41.6 | $CH_2$ | 1.65, m; 1.55, m |
| | 27 | 24.5 | CH | 1.20, m | | 59 | 19.6 | $CH_2$ | 1.66, m; 1.51, m |
| | 28 | 22.9 | $CH_3$ | 0.63, d (6.5) | | 60 | 14.6 | $CH_2$ | 0.92, t (7.0) |
| | 29 | 22.4 | $CH_3$ | 0.70, d (6.5) | | 47-OH | | | 6.48, d (4.7) |
| | 25-NH | | | 9.83, d (7.9) | | 52-OH | | | 5.64, d (5.8) |
| | | | | | | 57-OH | | | 5.69, d (5.8) |
| Ser | 30 | 171.7 | C | | | | | | |
| | 31 | 55.6c | CH | 5.17, m | | | | | |
| | 32 | 63.0 | $CH_2$ | 4.14, m; 4.07, m | | | | | |
| | 31-NH | | | 7.86, d (8.8) | | | | | |
| | 32-OH | | | 6.17, m | | | | | |

[a] Data recorded at 298 K, 600 MHz ($^{1}$H) and 150 MHz ($^{13}$C). [b] Assignments supported by 2D NMR.

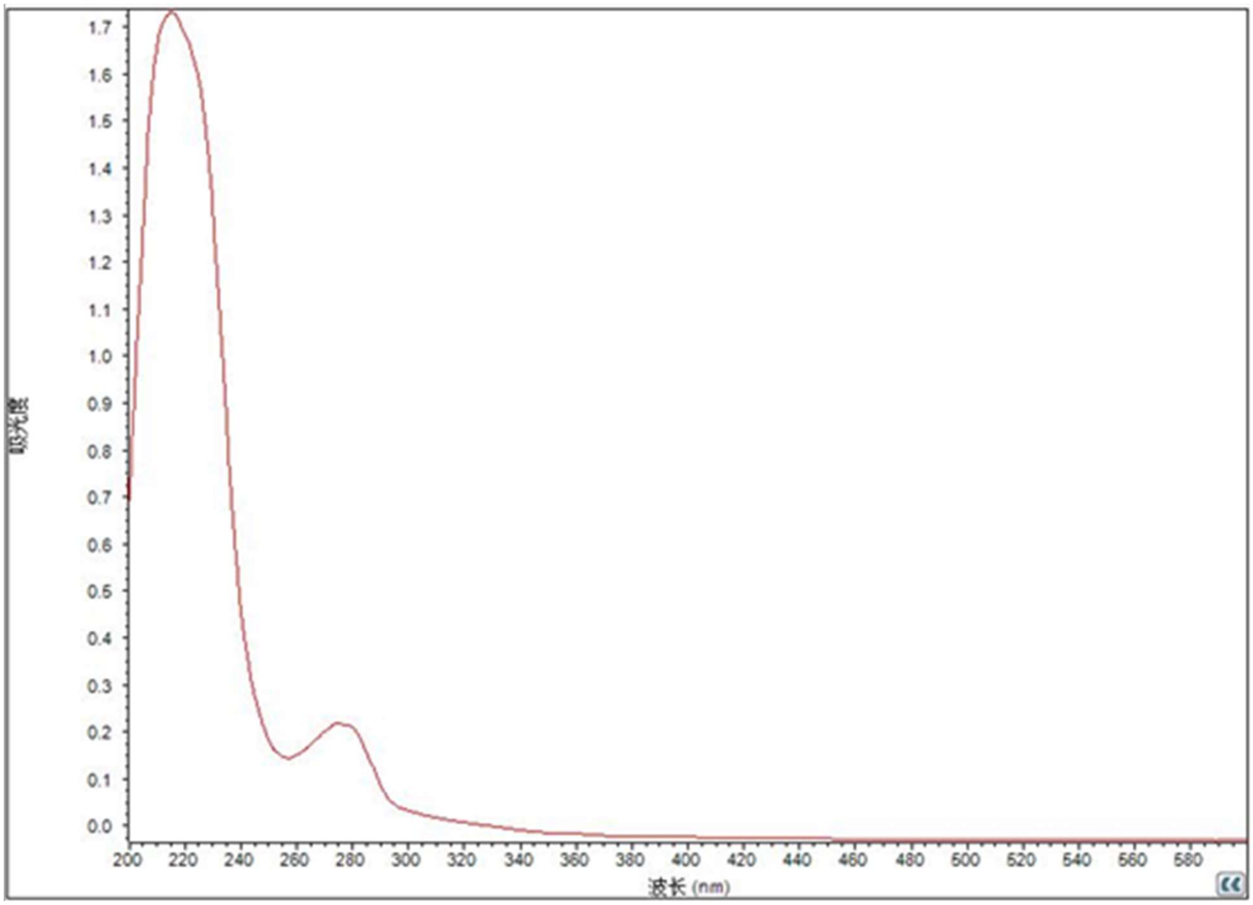


**Figure S11.** UV spectrum of **1** in MeOH at 0.25 mg/mL.

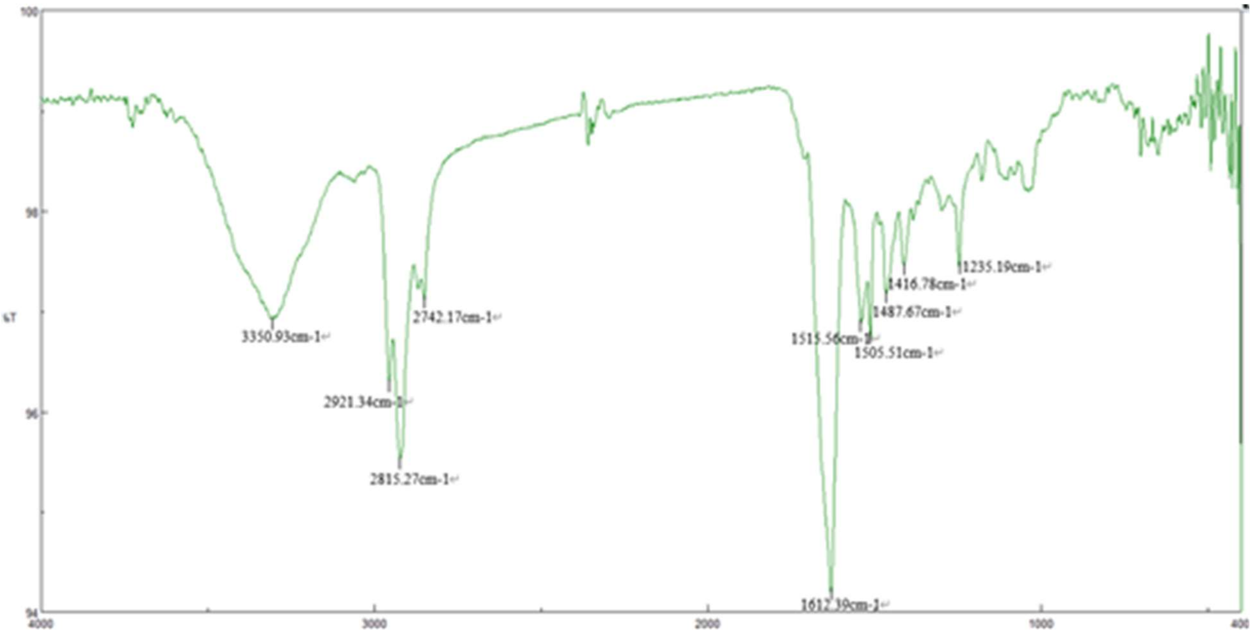


**Figure S12.** IR spectrum of **1** in KBr.

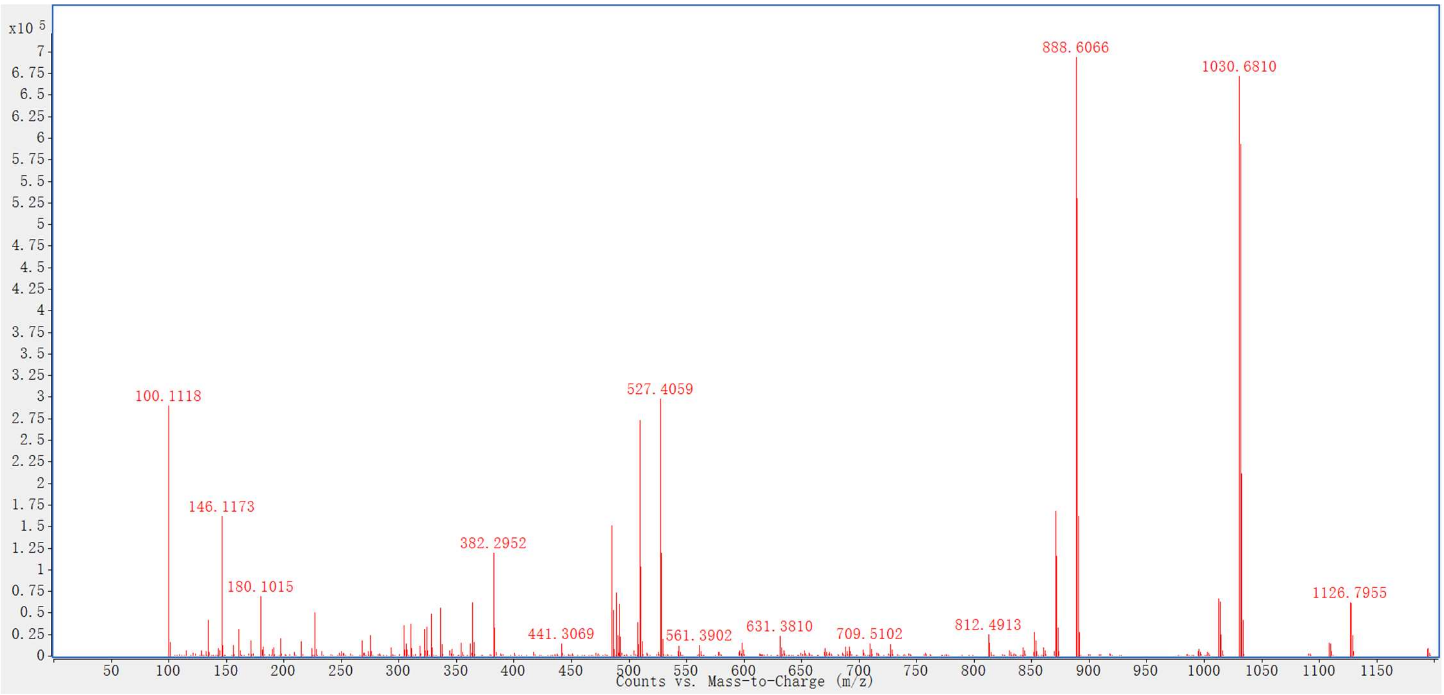


**Figure S13.** HRESIMS/MS of **1**.

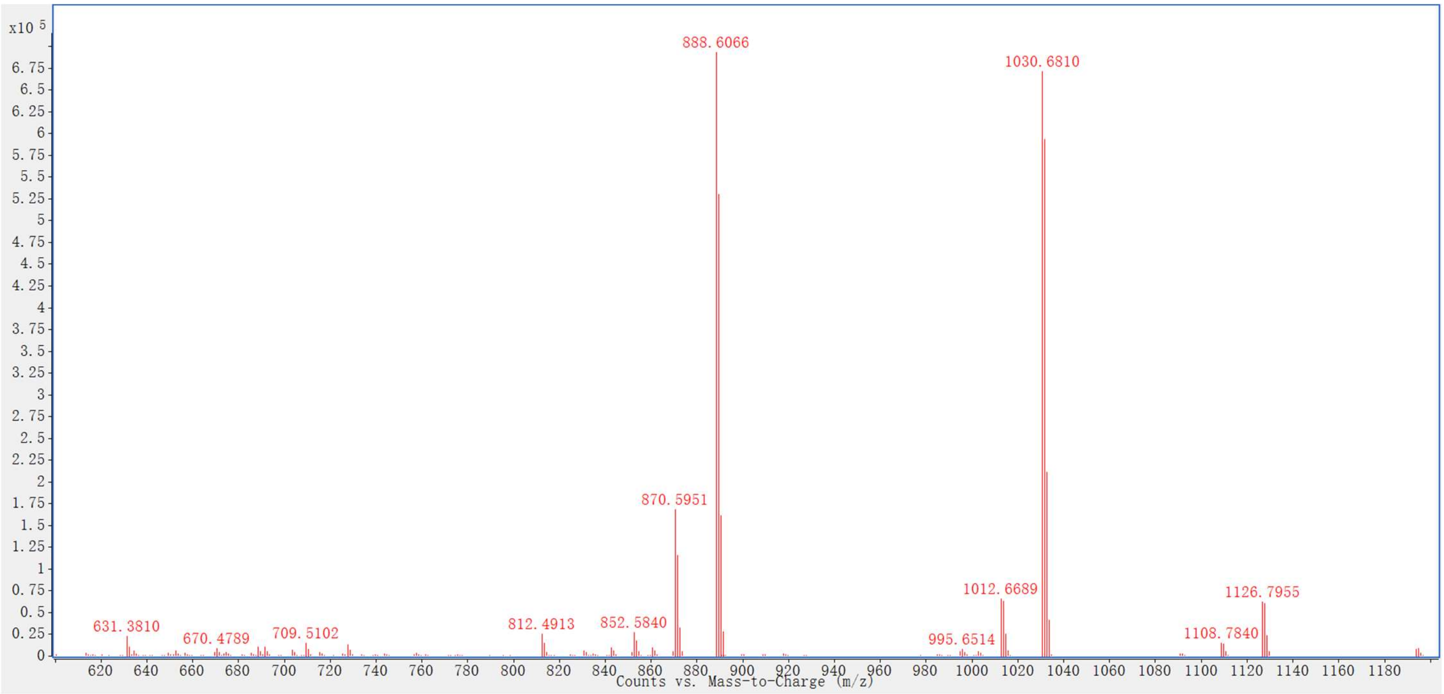


**Figure S14.** HRESIMS/MS spectrum of **1** (expanded view *m*/*z* 600-1200).

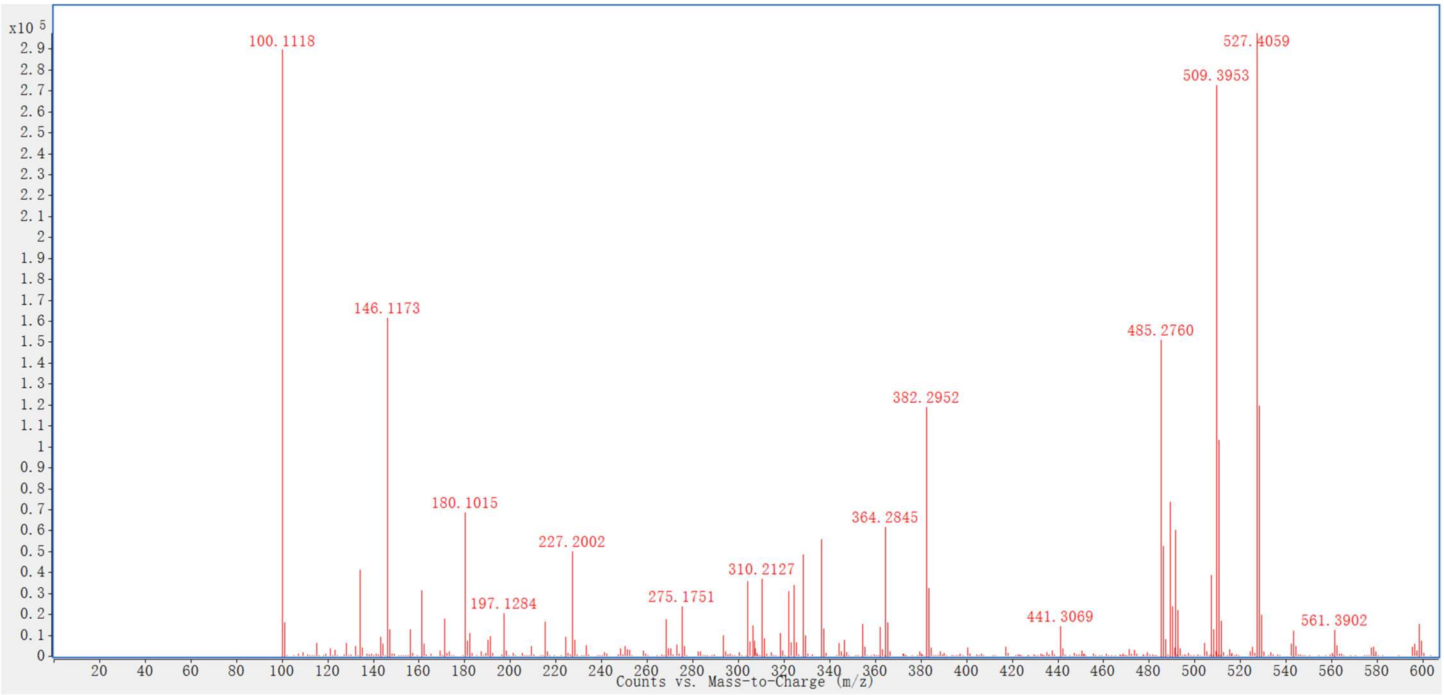


**Figure S15.** HRESIMS/MS spectrum of **1** (expanded view *m*/*z* 0-600).

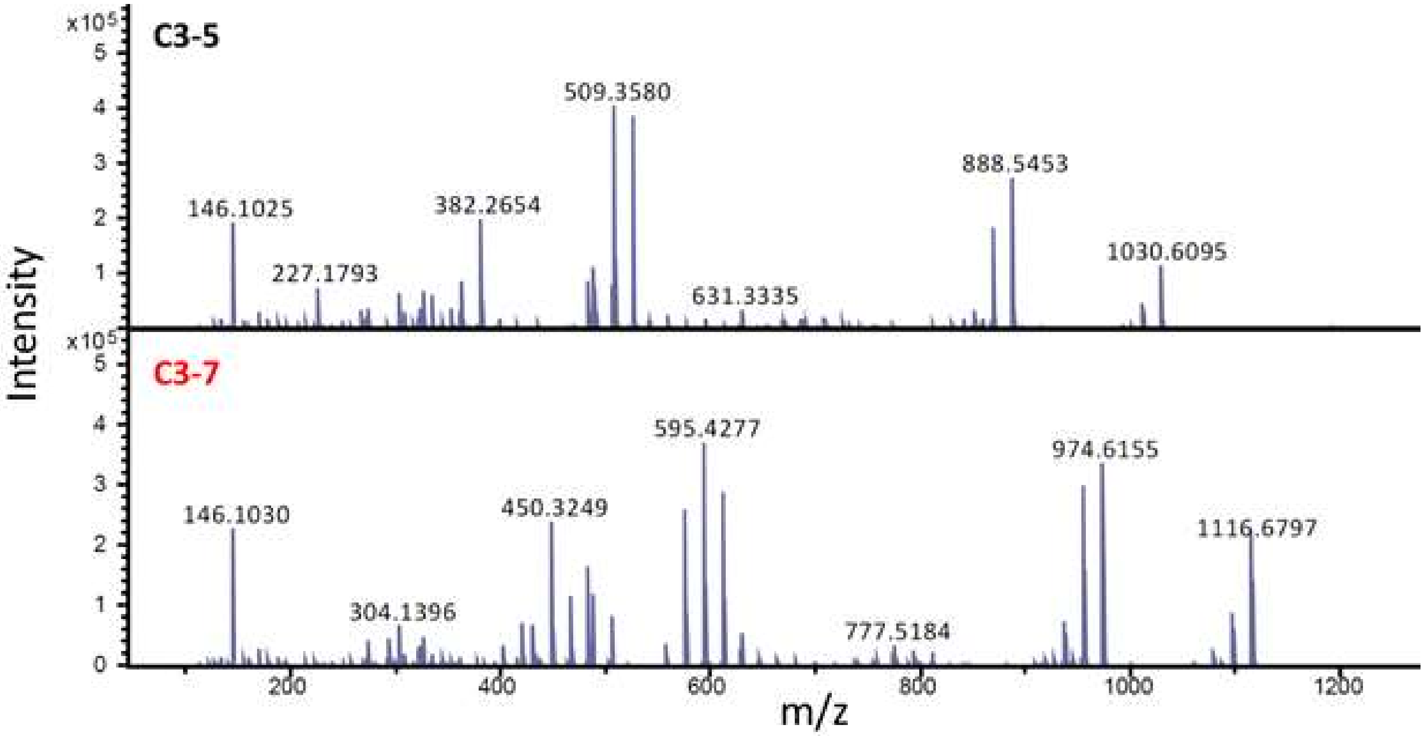


**Figure S16.** MS/MS spectra of wenchangamide A (**1;** *m*/*z* 1211) in C3-5 and wenchangamide B (proposed; *m*/*z* 1297) in C3-7.

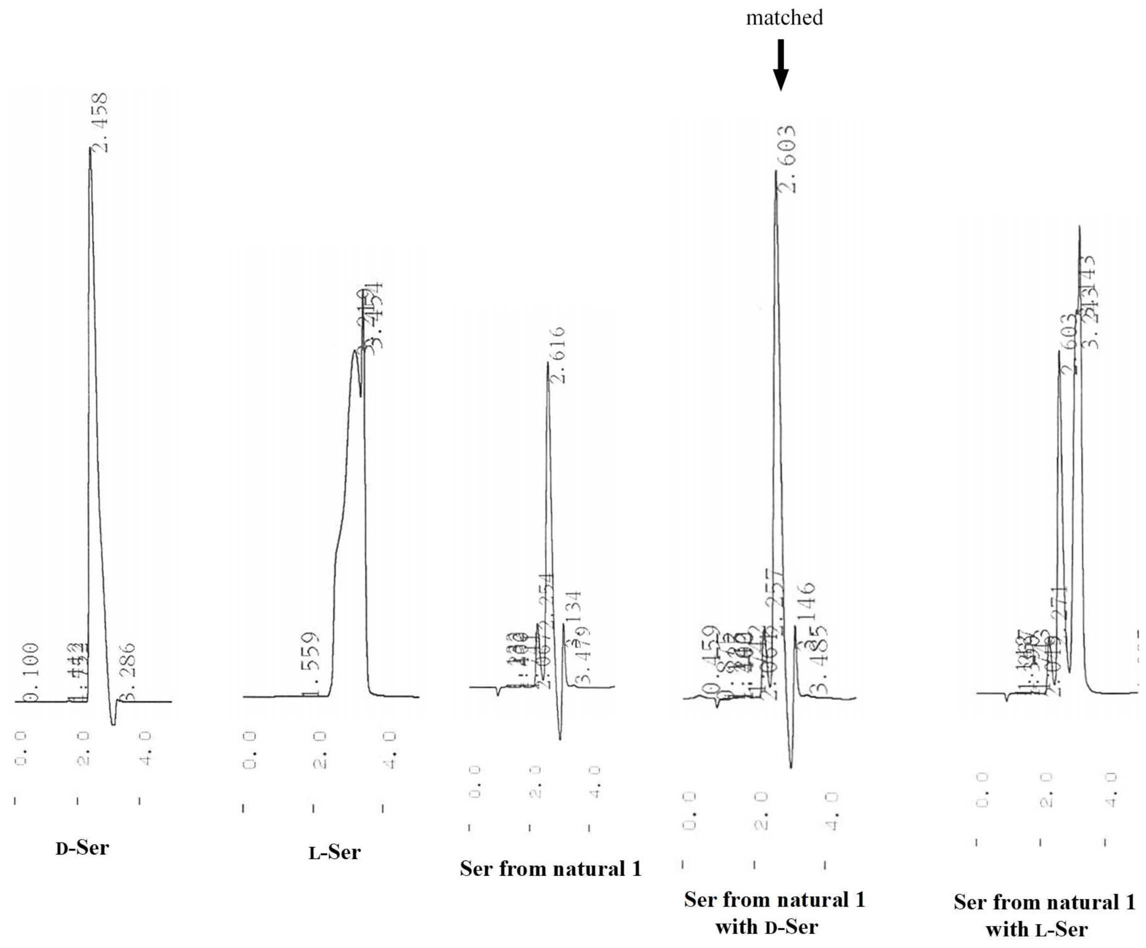


**Figure S17.** Chiral HPLC analysis of hydrosylates of **1** (Ser).

Ser: column, DAICEL CHIRALPAK MA(+) (   4.6 × 50 mm × 2 in series); flow rate 1 mL/min; detection, UV 254 nm; solvent 2.0 mM $CuSO_4$

$t_R$ (min): Authentic samples D-Ser (2.5), L-Ser (3.2)

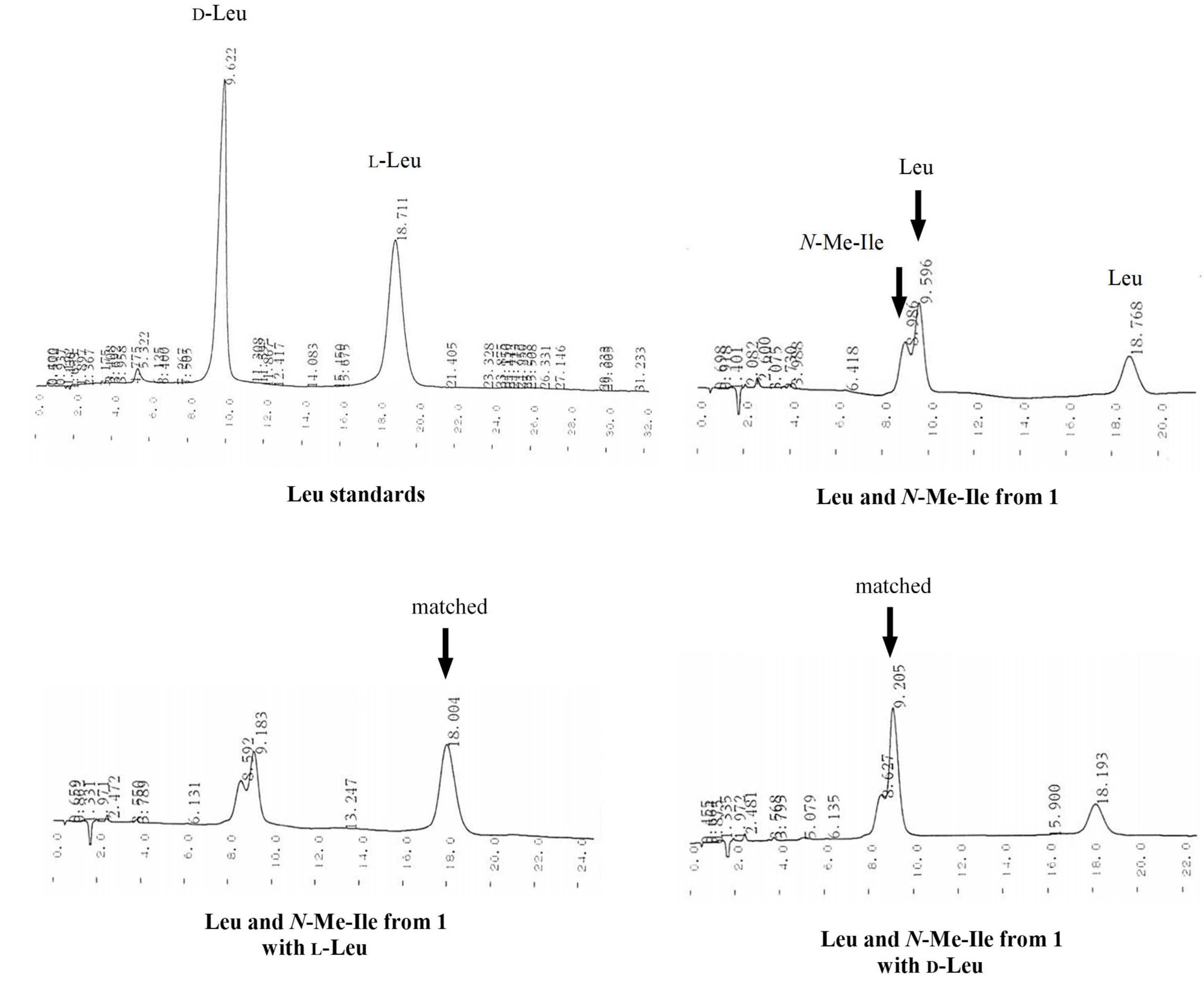


**Figure S18.** Chiral HPLC analysis of hydrosylates of **1** (Leu).

Leu: column, DAICEL CHIRALPAK MA(+) ( 4.6 × 50 mm); flow rate 1 mL/min; detection, UV 254 nm; solvent 2.0 mM $CuSO_4$

$t_R$ (min): Authentic samples D-Leu (9.6), L-Leu (18.7)

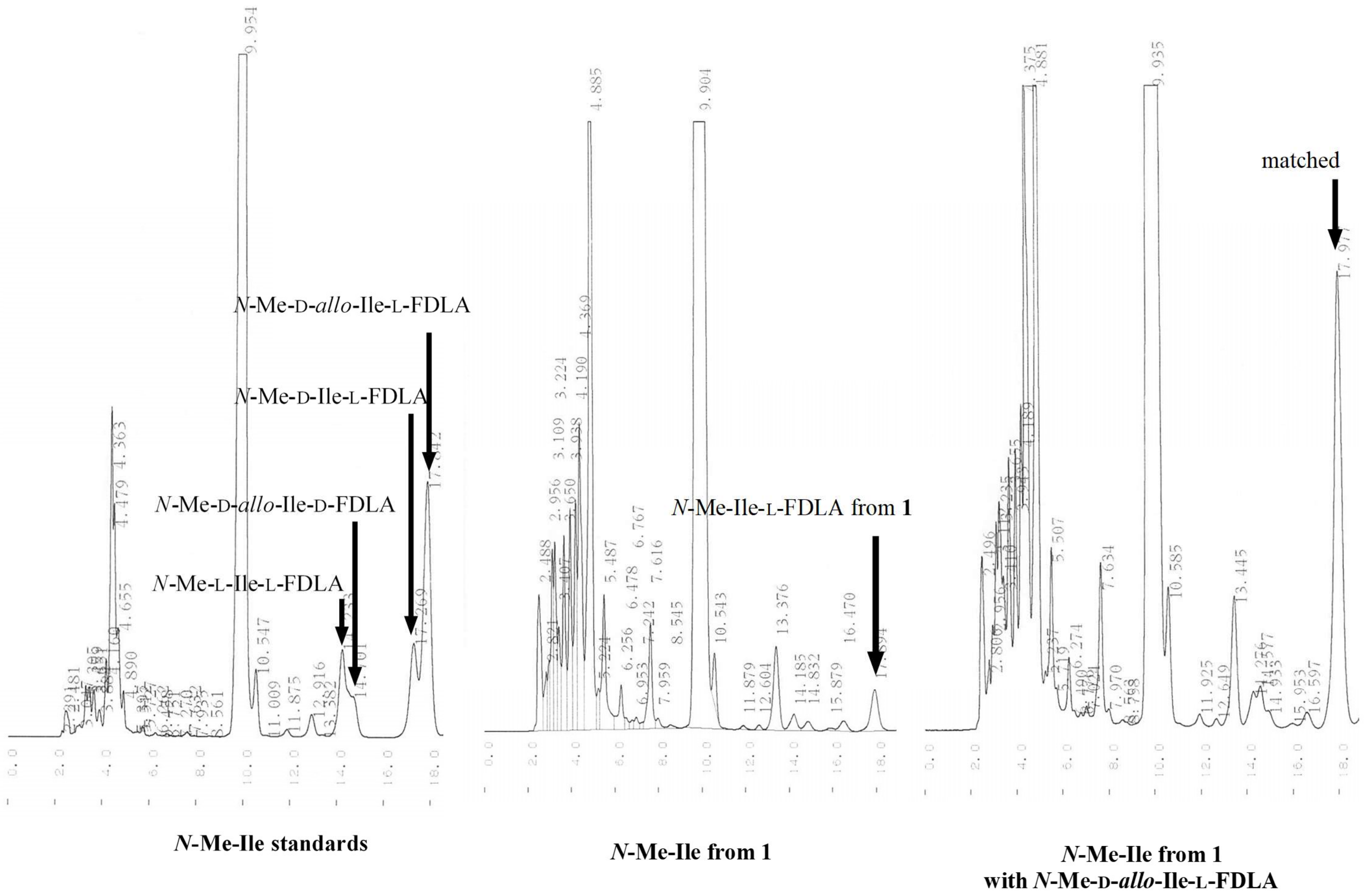


**Figure S19.** Chiral HPLC analysis of hydrosylates of **1** (*N*-Me-Ile).

*N*-Me-Ile: column, Cosmosil PBr ( 4.6 × 250 mm); flow rate 1 mL/min; detection, UV 340 nm; solvent 55% MeCN, 0.1% TFA

$t_R$ (min): Authentic samples *N*-Me-L-Ile-L-FDLA (14.2), *N*-Me-D-Ile-L-FDLA (17.3), *N*-Me-D-*allo*-Ile-D-FDLA (14.7), *N*-Me-D-*allo*-Ile-L-FDLA (17.8)

*N*-Me-D-*allo*-Ile-D-FDLA was used as a substitute of *N*-Me-L-*allo*-Ile-L-FDLA

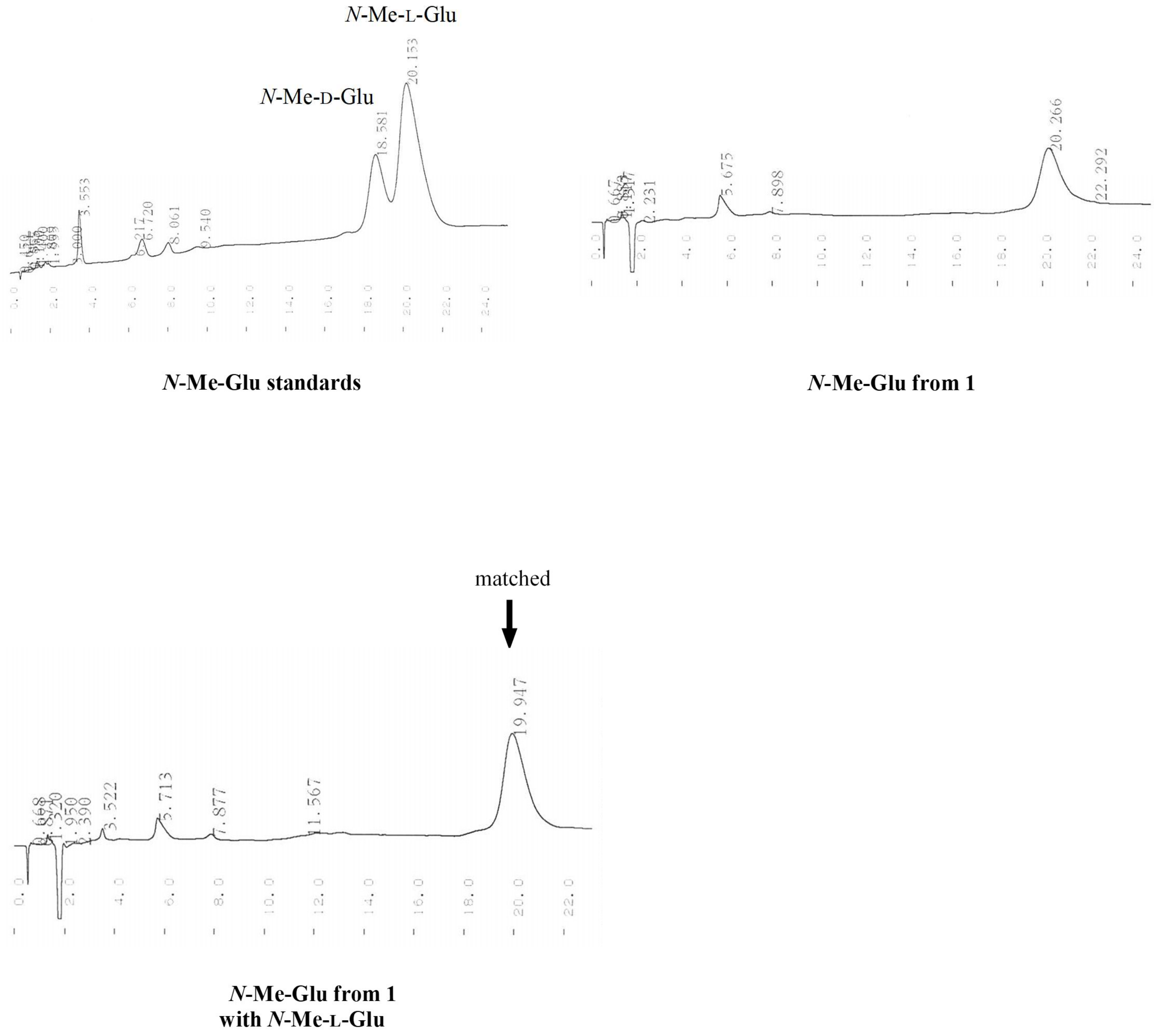


**Figure S20.** Chiral HPLC analysis of hydrosylates of **1** (*N*-Me-Glu).

*N*-Me-Glu: column, DAICEL CHIRALPAK MA(+) ( 4.6 × 50 mm); flow rate 1 mL/min; detection, UV 254 nm; solvent 2.0 mM $CuSO_4$

$t_R$ (min): Authentic samples *N*-Me-D-Glu (18.6), *N*-Me-L-Glu (20.2)

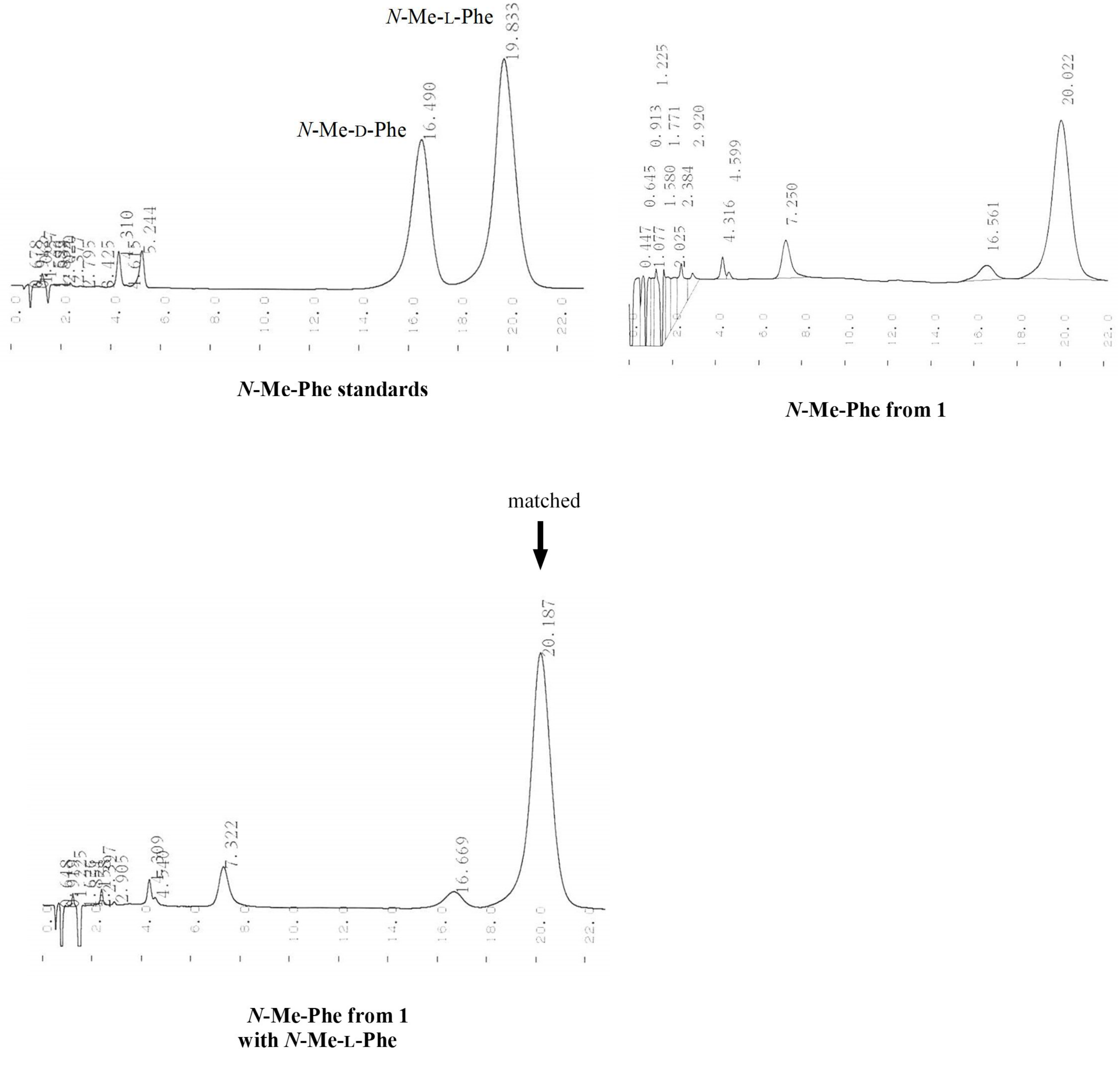


**Figure S21.** Chiral HPLC analysis of hydrosylates of **1** (*N*-Me-Phe).

*N*-Me-Phe: column, DAICEL CHIRALPAK MA(+) ( 4.6 × 50 mm); flow rate 1 mL/min; detection, UV 254 nm; solvent 2.0 mM $CuSO_4$

$t_R$ (min): Authentic samples *N*-Me-D-Phe (16.5), *N*-Me-L-Phe (19.8)

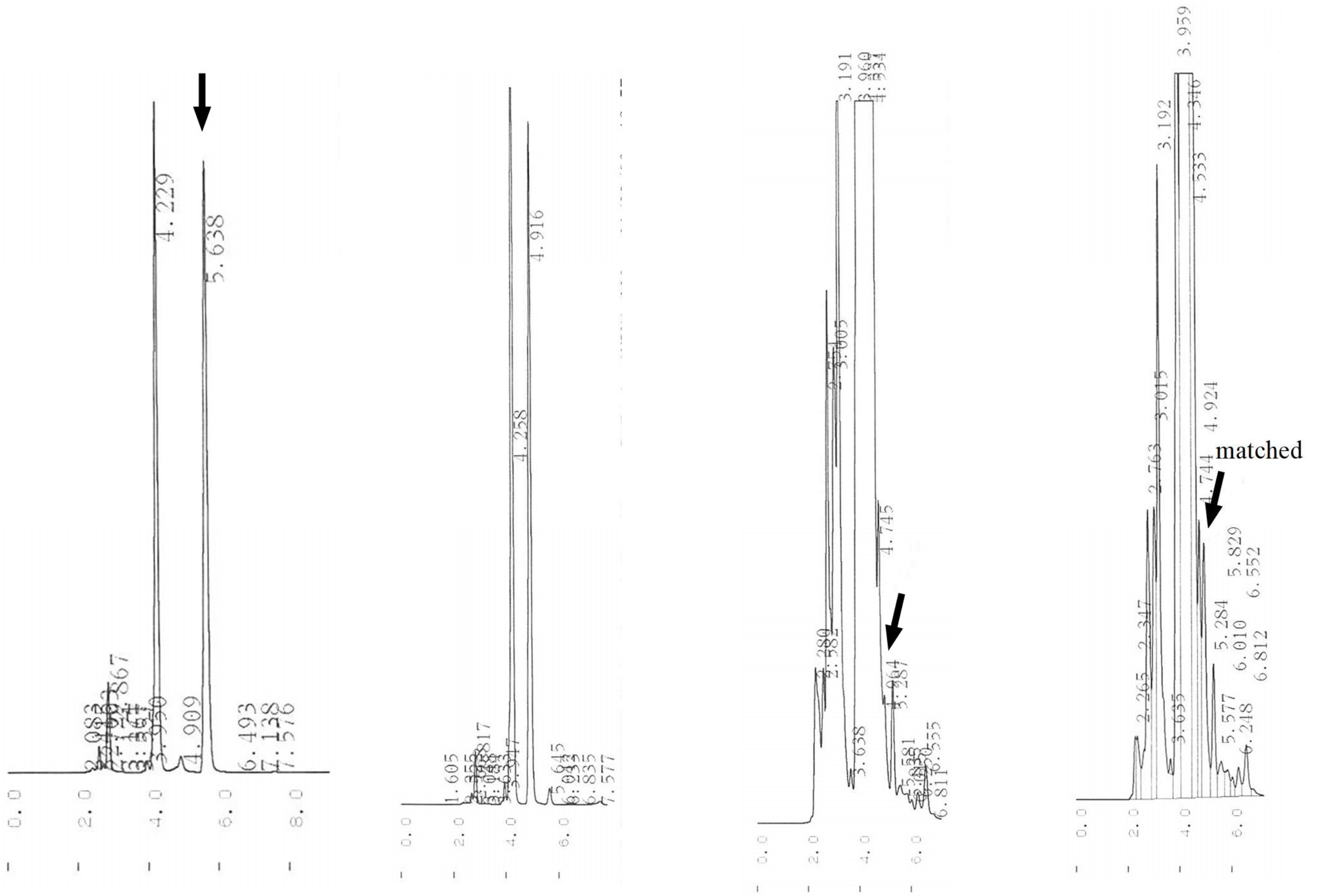


**(*S*)-AMP-D-FDLA** **(*S*)-AMP-L-FDLA** **AMP-L-FDLA from 1** **AMP-L-FDLA from 1 with (*S*)-AMP-L-FDLA**

**Figure S22.** Chiral HPLC analysis of hydrosylates of **1** (AMP).

AMP: column, Cosmosil Cholester ( 4.6 × 250 mm); flow rate 1 mL/min; detection, UV 340 nm; solvent 70% aqueous MeCN, 0.1% TFA

$t_R$ (min): Authentic samples (*S*)-AMP-D-FDLA (5.6), (*S*)-AMP-L-FDLA (4.9)

A

B

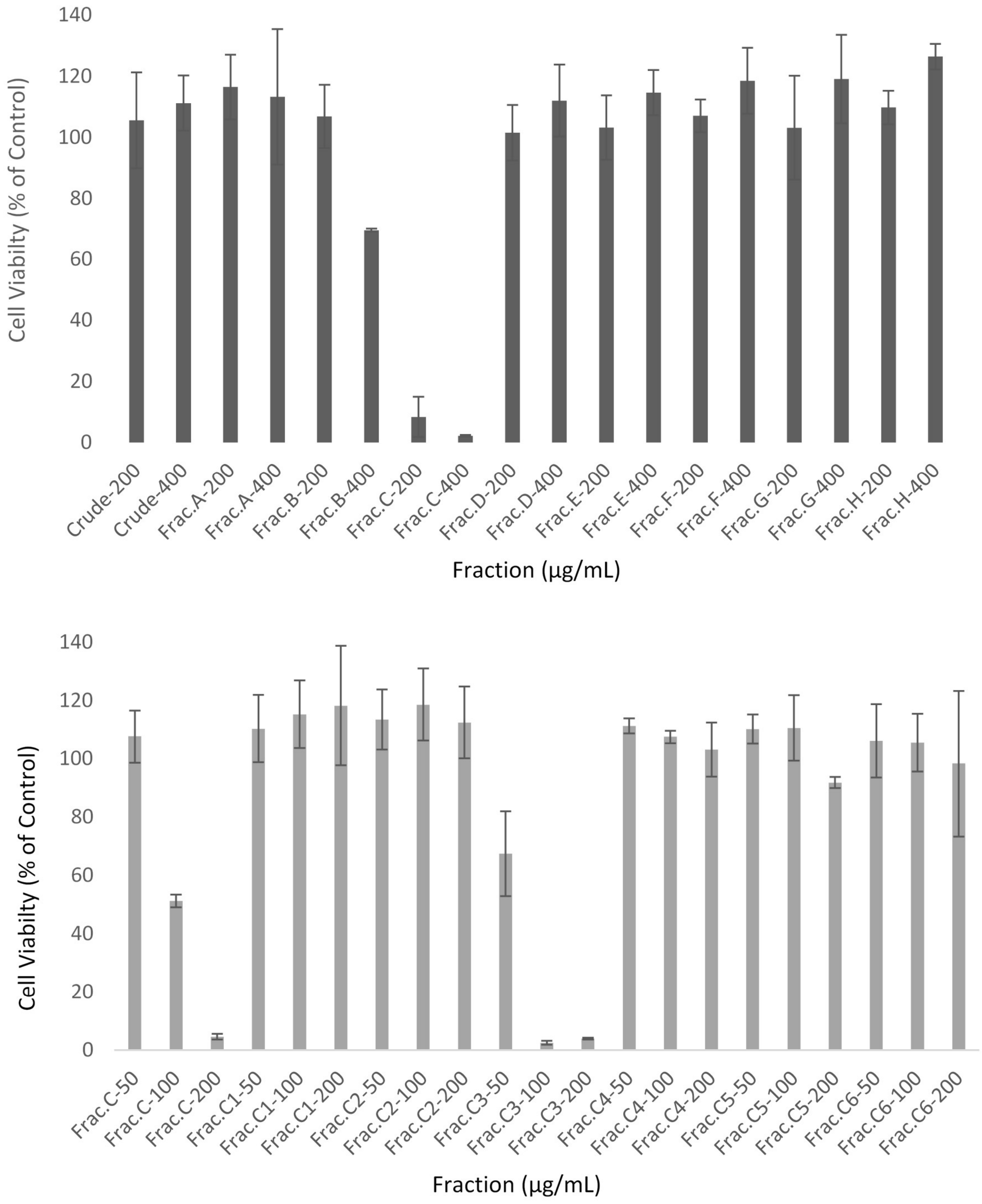


**Figure S23.** Bioactivity data used to guide fractionation of cf. *Neolyngbya* sp. extract using HCT116 human colorectal cancer cell viability *in vitro*.

Cells were untreated (control) and treated with (A) crude fractions or (B) sub-fractions for 24 h followed by cell viability assessment using XTT assay. Data represent the mean cell proliferation of three trials using the XTT assay, and bars represent the standard deviations.

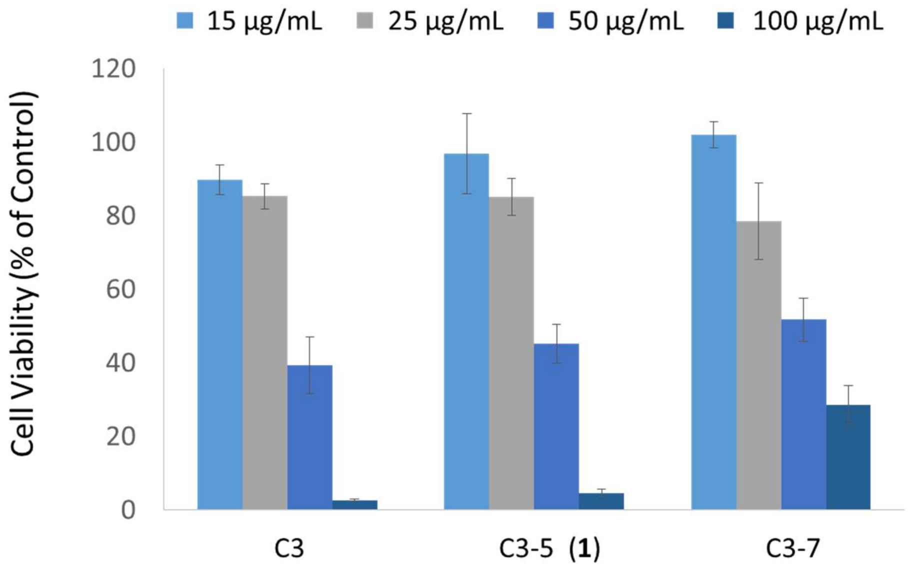


**Figure S24.** The *in vitro* effect of C3, C3-5 (compound **1**), and C3-7 on HCT116 cells viability, following 24 h of treatment with escalating concentrations.

Cell viability assessment done using the XTT assay. Results are presented as the percentage of control untreated HCT116 cells. Error bars represent the standard deviations.

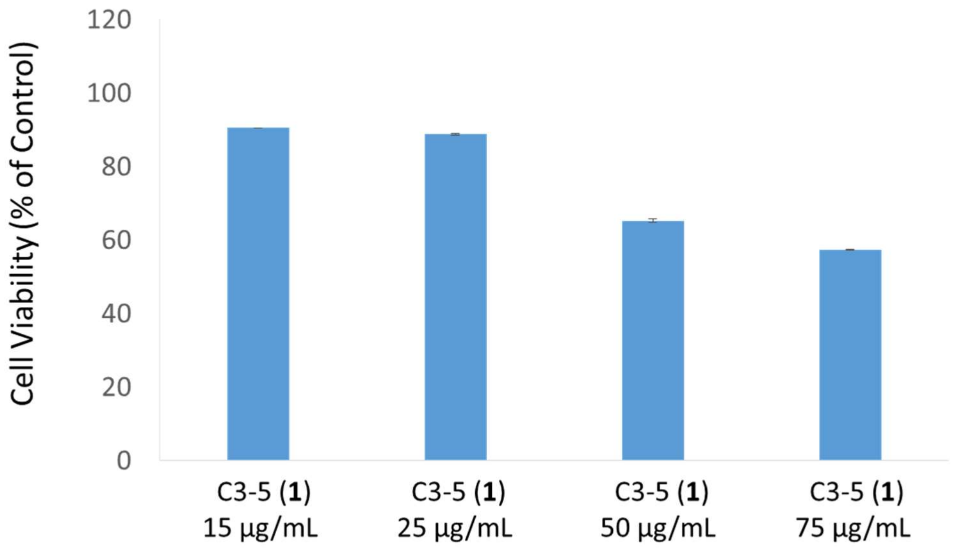


**Figure S25.** The *in vitro* effect of C3-5 (compound **1**) on HCT116 cells viability, following 8 h of treatment with escalating concentrations.

Cell viability assessment of C3-5 using the XTT assay. Results are presented as the percentage of control untreated HCT116 cells. Error bars represent the standard deviations.

https://gnps.ucsd.edu/ProteoSAFe/status.jsp?task=f62b23918fb24bca9f4a234f3555df50

**Weblink S1**. GNPS metabolomics data used to prepare main text Figure 4.

https://gnps.ucsd.edu/ProteoSAFe/status.jsp?task=0e36af9bc15d4d6c901292d5be8ff32b

**Weblink S2**. GNPS metabolomics data used to prepare main text Figure 9.

UGGGGAAUUUUCCGCAAUGGGCGAAGCCUGACGGAGCAAGACCGCGUGGGGGAGAAAGCCUUUGGGUUGUA
AACCCCUUUUCUGGGAAGAGUGACGGUACCAGGAAUCAGCCUCGGCUAACUCCGUGCCAGCAGCCGCGGUA
AUACGGAGGAGGCAGCGUUAUCCGGAAUGAUUGGGCGUAAAGCGUCCGCAGGUGGAAGUCGUGUUAAACCA
AGCUAACUGAGGGCAUGGAAACUGCUGAGUCGGUAGGGGCAGAGGGAAUUCCUGGUGUAGCGGUGAAAUG
CGUAGAGAUCAGGAAGAACACCGUGGCGAAAGCGCUCUGCUGGGCCACUGACACUCAGGGACGAAAGCUAG
GGGAGCGAAUGGG

**Sequence S1.** 16S rRNA gene V3-V4 amplicon used to prepare main text Figure 3C.

This was the most prominent sequence obtained from the microbiome analysis of sample HAINAN-19SEP17-3.
